# Surface cholesterol-enriched domains specifically promote invasion of breast cancer cell lines by controlling invadopodia and extracellular matrix degradation

**DOI:** 10.1007/s00018-022-04426-8

**Published:** 2022-07-12

**Authors:** Mauriane Maja, Danahe Mohammed, Andra C. Dumitru, Sandrine Verstraeten, Maxime Lingurski, Marie-Paule Mingeot-Leclercq, David Alsteens, Donatienne Tyteca

**Affiliations:** 1 CELL Unit and PICT Imaging Platform, de Duve Institute, UCLouvain, B1.75.05, avenue Hippocrate, 75, 1200 Brussels, Belgium; 2 Louvain Institute of Biomolecular Science and Technology (LIBST), UCLouvain, Ottignies-Louvain-la-Neuve, Belgium; 3 Cellular and Molecular Pharmacology Unit (FACM), Louvain Drug Research Institute, UCLouvain, Brussels, Belgium

**Keywords:** Matrigel invasion, Cholesterol transversal asymmetry, Endocytosis, Membrane contact sites, MCF10A series, MDA-MB-231

## Abstract

Tumor cells exhibit altered cholesterol content. However, cholesterol structural subcellular distribution and implication in cancer cell invasion are poorly understood mainly due to difficulties to investigate cholesterol both quantitatively and qualitatively and to compare isogenic cell models. Here, using the MCF10A cell line series (non-tumorigenic MCF10A, pre-malignant MCF10AT and malignant MCF10CAIa cells) as a model of breast cancer progression and the highly invasive MDA-MB-231 cell line which exhibits the common TP53 mutation, we investigated if cholesterol contributes to cancer cell invasion, whether the effects are specific to cancer cells and the underlying mechanism. We found that partial membrane cholesterol depletion specifically and reversibly decreased invasion of the malignant cell lines. Those cells exhibited dorsal surface cholesterol-enriched submicrometric domains and narrow ER-plasma membrane and ER-intracellular organelles contact sites. Dorsal cholesterol-enriched domains can be endocytosed and reach the cell ventral face where they were involved in invadopodia formation and extracellular matrix degradation. In contrast, non-malignant cells showed low cell invasion, low surface cholesterol exposure and cholesterol-dependent focal adhesions. The differential cholesterol distribution and role in breast cancer cell invasion provide new clues for the understanding of the molecular events underlying cellular mechanisms in breast cancer.

**Supplementary Information:**

The online version contains supplementary material available at 10.1007/s00018-022-04426-8.

## Introduction

Cell migration is a fundamental property of cancer and metastasis. Formation and growth of secondary tumor require cancer cells to navigate through diverse microenvironments, including extracellular matrix (ECM) and blood vessels, and to adapt to mechanical stresses applied to them. Cancer cell lines and cells derived from cancerous tissues exhibit an increased deformability as compared to their healthy counterparts [1–4]. Cancer cells are able to adapt their morphology and invasiveness by remodeling their cytoskeleton in connection with their nanomechanical properties [5]. However, the contribution of the plasma membrane (PM) lipid content and biophysical properties in cancer cell invasion is poorly understood [6, 7].

Due to aberrant lipid metabolism, cancer cells exhibit an altered PM lipid profile as compared to their healthy counterparts [6]. Although some common features can be highlighted, including ceramide loss and phosphatidylserine (PS) surface exposure [8–12], membrane lipid alterations are generally tumor-specific and even subtype-specific. For instance, the human MT3 breast cancer cell line exhibits an increase in mono- and polyunsaturated fatty acid content which correlates with metastasis [13]. Lipidomic analyses of the highly invasive MDA-MB-231 cell line have shown that PS, phosphatidylglycerol and phosphatidic acid are upregulated while sphingomyelin (SM) is downregulated as compared to the poorly invasive MCF7 cell line [14]. In contrast, using the MCF10A cell line series, Vidavsky et al. evidenced that SM is increased while phosphatidylethanolamine (PE) and phosphatidylcholine (PC) are less abundant in invasive cells compared to precancerous cultures [15]. In breast cancer samples, the absence of estrogen receptors is associated with upregulated SM, PC, PE and phosphatidylinositol species and specific PC species correlate with more aggressive breast tumors and poorer overall survival [16].

Cholesterol (chol) content is also generally described as altered in breast cancer. For instance, higher expression of chol biosynthesis genes has been shown to associate with a worse prognosis of basal-like breast cancer patients [17]. Multidrug-resistant cells exhibit higher chol that turns the PM more rigid and, thus, less permeable for drugs [18]. On the other hand, lower chol levels in metastatic cells correlate with a more deformable PM, increasing its invading capacity [13, 19]. Nevertheless, it is not known whether PM chol distribution is also deregulated in breast cancer and how. The difficulties could result from the lack of appropriate chol visualization tools and of high-resolution live microscopy approaches requested to analyze lipids, but also from the limited availability of isogenic cell models to evaluate the specificity of the effects. We recently used atomic force microscopy (AFM) combined with chol vital imaging by the domain 4 (D4) of the bacterial toxin perfringolysin O coupled to mCherry (hereafter named Theta toxin fragment [20–22]) on the MCF10A cell line series [23]. This model includes three human mammary cell lines, which offer the same background but increasing degrees of malignancy: MCF10A (A, non-tumorigenic), MCF10AT (AT, pre-malignant) and MCF10CAIa (CAIa, malignant) [24]. We showed that, while the cytoskeleton of the malignant CAIa cell line is, as expected, softer than normal A cells, its PM is surprisingly stiffer and correlates with higher surface chol content and cell malignancy [23].

In the present study, we used the same series of breast cell lines to investigate (1) if surface chol contributes to cancer cell invasion, (2) whether the effects are specific to cancer cells, opening the possibility to specifically target this lipid in cancer, and (3) the underlying mechanism. To then test for the relevance of our observations, the study was extended to the triple-negative breast cancer cell line, MDA-MB-231, which exhibits the common TP53 mutation and is highly invasive [25]. We showed that the surface chol was specifically required for invasion of malignant CAIa and MDA-MB-231 cells. This surface chol was distributed in submicrometric domains that can be endocytosed and reach the cell ventral face where they were involved in the formation of invadopodia, allowing for gelatin degradation. Our data help to better understand the contribution of membrane chol to breast cancer cell invasion.

## Materials and methods

### Antibodies and reagents

Rabbit anti-Vimentin (D21H3, immunofluorescence IF 1:100, western blotting WB 1:100) and rabbit anti-Cortactin (H222, IF 1:200, WB 1:1000) were from Cell Signaling Technology. Mouse anti-Paxillin (5H11, IF 1:200, WB 1:1000) and TopFluor^®^ Cholesterol (810255P) were from Merck Millipore. Mouse anti-KDEL (10C3, IF 1:300), Dyngo^®^ 4a (ab120689) and Hoechst 33258 (ab228550) were from Abcam. Alexa Fluor™ 568 Phalloidin (A12380), Alexa Fluor™ 647 Phalloidin (A22287), Oregon Green™ 488 conjugate (G13186), Fluo4-AM (F-14201), ER-Tracker™ Red (E34250), LysoTracker™ Green DND-26 (L7526), LysoTracker™ Red DND-99 (L7528), Alexa Fluor 568 Transferrin (T23365), DAPI (D3571) and fluorescent secondary antibodies were from ThermoFisher Scientific. Peroxidase-conjugated secondary antibodies were from ThermoFisher Scientific and Sigma. SiR-actin (SC001) was from Spirochrome. GM6001 MMP inhibitor (BML-EI300-0001) was from Enzo Life Sciences. Mouse anti-α-Tubulin (DM1a, IF 1:100, WB 1:1000), methyl-β-cyclodextrin (mβCD; C4555), cholesterol-Water Soluble (C4951), cytochalasin D (cytoD) from Zygosporium mansonii (C8273) and growth factor-reduced ECM gel from Engelbreth-Holm-Swarm mouse sarcoma (E6909) were from Sigma-Aldrich.

### Expression and purification of Theta toxin fragment

The expression plasmid pET28/His-mCherry-Theta-D4 encodes for an N-terminal 6xHis-tag followed by the monomeric red fluorescent protein mCherry and the C-terminal non-toxic domain D4 of the chol-specific Theta toxin (Theta-D4). The plasmid was generated by swapping Dronpa for mCherry from pET28/His-Dronpa-theta-D4 [26]. Expression, purification, biochemical characterization and storage of Theta toxin fragment were performed as described in [20].

### Cell line culture and chemical treatments

MCF10A, AT and CAIa cell lines were grown as previously described with the addition of 10 mM Hepes in the media [23]. MDA-MB-231 were grown in DMEM supplemented with 10% Fetal Bovine Serum (FBS), penicillin (100 U mL^−1^) and streptomycin (100 µg mL^−1^) at 37 °C with 5% CO_2_. To partially deplete chol, cells were pre-incubated in a serum-free medium containing 1–6 mM mβCD for 2 h at 37 °C. To replete chol, depleted cells were subsequently incubated in serum-free medium containing mβCD charged with chol in a (6:1) molar ratio for 1 h. To inhibit MMP activity, cells were maintained during the whole experiment in a serum-free medium containing 10 µM GM6001 at 37 °C. To inhibit actin polymerization, cells were pre-incubated in a serum-free medium containing 0.5 µM cytoD for 2 h at 37 °C. To inhibit endocytosis, cells were preincubated in a serum-free medium containing 20 µM Dyngo4a for 30 min at 37 °C. The drug was maintained during the whole experiment. To check for the efficiency of mβCD and maintenance of effect during the time needed for an invasion assay despite agent removal, the total cell chol content was extracted and assessed with an Amplex Red cholesterol assay kit (Invitrogen) exactly as previously described [23].

### Matrigel invasion assay

ECM Gel was diluted in DMEM to a final concentration of 0.5 or 2 mg mL^−1^. 140 µL were then added to the ThinCert™ Tissue Culture Inserts (8 µm pore size, Greiner) and allowed to polymerize overnight at 37 °C. Excess of ECM Gel was removed by vacuum and 3.5 × 10^4^ cells were seeded on top of the ECM Gel-coated insert and allowed to adhere overnight. Cells were either serum-starved or pre-treated with 2 mM mβCD in combination or not with 0.5 µM cytoD for 2 h. Alternatively, they were repleted with chol for 1 h. Bottom chamber was filled with serum-free medium (background control) or 10% serum-containing medium (to assess total invasion) and cells were allowed to invade the ECM Gel for 6–12 h or 24 h. For the latter condition, cell proliferation was inhibited by 30 µM Mitomycin C (Sigma). Invading cells were fixed in 4% paraformaldehyde (PAF) for 20 min, stained with crystal violet for 10 min and washed with phosphate buffer saline (PBS). Non-invading cells were wiped from the upper chamber with a cotton swab. Ten images per insert were captured with a wide-field fluorescence microscope Observer.Z1 (20 × objective). To quantify the number of invading cells per condition, the total area of invading cells was determined by the ZEN 2.6 software (Blue edition, Zeiss) and was then divided by the area of a single cell manually surrounded. The invasion was calculated by subtracting the background control from the total number of invading cells.

### AFM Young’s Modulus measurements and data analysis

AFM images were acquired using an atomic force microscope (Bioscope Resolve, Bruker) operated in the PeakForce QNM mode (Nanoscope software v9.2) and coupled to an inverted epifluorescence microscope (Zeiss Observer Z.1). A 40 × oil objective (NA = 0.95) was used. The AFM was equipped with a 150 μm piezoelectric scanner and a cell-culture chamber allowing to control the temperature, the humidity and the CO_2_ concentration [27]. Overview images of cell surfaces (~ 30 μm^2^) were recorded at imaging forces of 500 pN using PFQNM-LC probes (Bruker) having tip lengths of 17 μm, tip radii of 65 nm and opening angles of 15°. The spring constant of the cantilevers was calibrated with a vibrometer (OFV-551 Polytec, Waldbronn) by the manufacturer. The pre-calibrated spring constant was used to determine the deflection sensitivity using the thermal noise method before each experiment [28]. The AFM tip was oscillated in a sinusoidal fashion at 0.25 kHz with a 750 nm amplitude in the PeakForce Tapping mode. The sample was scanned using a frequency of 0.2 Hz and 128 pixels per line. AFM images were analyzed and Young’s modulus values of elasticity were extracted as previously described [23].

### Live cell imaging of cholesterol, actin, calcium, endoplasmic reticulum, lysosomes and transferrin and quantification of surface cholesterol

Coverslips were coated with 20 µg mL^−1^ fibronectin (Sigma-Aldrich) overnight at 4 °C or left uncoated. Cells were grown on coverslips overnight, washed 2 times with DMEM/F12 at room temperature and then labeled. For chol labeling, cells were incubated with 1.5 µM mCherry-Theta toxin fragment in fatty acid-free Bovine Serum Albumin (1 mg mL^−1^) for 20 min at the indicated temperature, as previously described [20], or incubated in serum-free medium containing 15 µM mβCD charged with 5 µM TopFluor-Cholesterol in a (3:1) molar ratio for 20 min at 4 °C. For actin labeling, cells were incubated with 1 µM SiR-actin probe for 25 min at 37 °C. For calcium (Ca^2+^) labeling, cells were incubated with 1 µM Fluo4-acetoxymethyl (AM) ester for 1 h at 37 °C then incubated in serum-free medium for a further 30 min at 37 °C to allow for de-esterification of AM esters. For ER and lysosomes labeling, cells were respectively incubated with 1 µM ER-Tracker and 50–100 nM LysoTracker probe for 30 min at 37 °C. The latter probe was maintained during the whole experiment, including the washing step. For fluorescent Transferrin endocytosis tracking, cells were incubated with 20 µg mL^−1^ Alexa 568-transferrin for 10 min at 37 °C. Labeled cells were then washed 2 times with DMEM/F12 at room temperature. Coverslips with labeled living cells were placed in medium-filled LabTek chambers and observed with a Zeiss Cell Observer Spinning Disk (COSD) confocal microscope using a plan-Apochromat 63 × NA 1.4 water immersion objective. X–Y confocal images and/or z-stacks were taken. Chol distribution at the top of the cells was quantified on z-stacks using ImageJ/Fiji. Images were converted to 8-bit grayscale and the mean grey value of the background was subtracted from the mean grey value of Theta fluorescence at the top section of each cell. Data are presented as Theta dorsal fluorescence intensity.

### Cell immunofluorescence and quantification of focal adhesions, invadopodia, endoplasmic reticulum spreading, ER-Lysosomes contacts and apoptosis

Cells were seeded on glass or fibronectin-coated coverslips, washed with PBS, fixed with 4% PAF for 30 min, washed with PBS 5 times for 5 min, permeabilized with 0.1% Triton X-100 in PBS for 15 min and blocked with 5% Normal Goat Serum in PBS for 1 h, all at room temperature. Fixed cells were then immunolabeled with primary antibodies overnight at 4 °C, washed with PBS 3 times for 5 min, stained with fluorescent secondary antibody or Alexa Fluor™ Phalloidin and Hoechst 33258 for 1 h or DAPI for 5 min at room temperature in the dark, washed again with PBS 3 times for 5 min, mounted with Dako and examined with the COSD confocal microscope using a plan-Apochromat 100 × NA 1.4 oil immersion objective and the same settings for illumination. Quantification of focal adhesions number and size was done using ImageJ/Fiji [29]. In brief, confocal images were opened in grayscale (black and white) mode, the background was subtracted using a rolling ball algorithm, local contrast of the images was enhanced by running CLAHE (Contrast Limited Adaptative Histogram Equalization) plugin, background was further minimized by applying mathematical exponential (EXP) and a manual threshold was used to segment areas of focal adhesions based on areas positive for paxillin. Number of cells presenting focal adhesions was counted manually based on paxillin and Hoechst nuclear staining. For invadopodia, the brightness and contrast of confocal images were adjusted manually to visualize invadopodia double positive for actin and cortactin and the area of invadopodia was manually surrounded by the generated images. Number of cells presenting invadopodia was counted manually based on actin, cortactin and Hoechst nuclear staining. Quantification of ER spreading through the cytoplasm was done using ImageJ/Fiji. Images were converted to 8-bit grayscale and manual threshold was used to segment areas of ER and cytoplasm based on KDEL and α-Tubulin labeling, respectively. Data are presented as difference between cytoplasm and ER areas. Contacts between LysoTracker^+^ structures and ER were quantified based on fluorescence intensity profiles. Each peak of LysoTracker signal was assigned a score of 0 or 1 (0 = no contact with ER signal peak; 1 = close contact with ER signal peak). Data are expressed as a percentage of close contacts between LysoTracker^+^ structures and ER on total LysoTracker^+^ structures. For apoptosis, number of apoptotic nuclei was counted manually based on morphology and DAPI staining and expressed in percentage of total nuclei.

### Gelatin degradation assay and quantification

Oregon Green™ 488 conjugate gelatin-coated coverslips were prepared as previously described [30]. Coverslips were equilibrated in serum-containing medium for 1 h at 37 °C before plating 1 × 10^5^ cells per coverslip. Cells were starved or treated for 2 h before incubation in serum-containing medium for 6–12 h to allow gelatin matrix degradation. Immunofluorescence staining was performed as described above and immunolabeled cells were examined with the COSD confocal microscope using a plan-Apochromat 63 × NA 1.4 water immersion objective and the same settings for illumination. Quantification of total gelatin degradation area per total cell area was done using ImageJ/Fiji [30]. In brief, confocal images were opened in grayscale (black and white) mode, background was subtracted using a rolling ball algorithm and manual threshold was used to segment areas of gelatin degradation based on black areas in the Oregon Green fluorescent gelatin *vs* total cell area based on Phalloidin labeling. Data are presented as total gelatin degradation area per total cell area.

### Statistical analyses

Statistical analyses were performed using GraphPad Prism Version 8.0.2. All data are expressed as means of *n* independent experiments ± SD. Statistical tests were performed only when *n* ≥ 3. Tests were non-parametrical when *n* ≤ 10. To compare the three cell lines and the effect of multiple treatments, parametrical ordinary one-way ANOVA followed by a suitable post-hoc comparison test or non-parametrical Kruskal–Wallis test followed by Dunn’s comparison test were performed. To compare two samples, parametrical unpaired t test or non-parametrical unpaired Mann–Whitney test were done. To compare the effect of one treatment with a hypothetical mean of 100% representing the control, parametrical one-sample t test or non-parametrical Wilcoxon signed-rank test were performed. Comparisons between two or more groups are indicated with bars on top of the graphs. Comparisons with the control value are indicated above the columns. ns not significant, *p < 0.05, **p < 0.01**,** ***p < 0.001, ****p < 0.0001.

## Results

### The higher invasion potential of malignant cells specifically depends on matrix metalloproteinase activity and cholesterol content

**Fig. 1:**
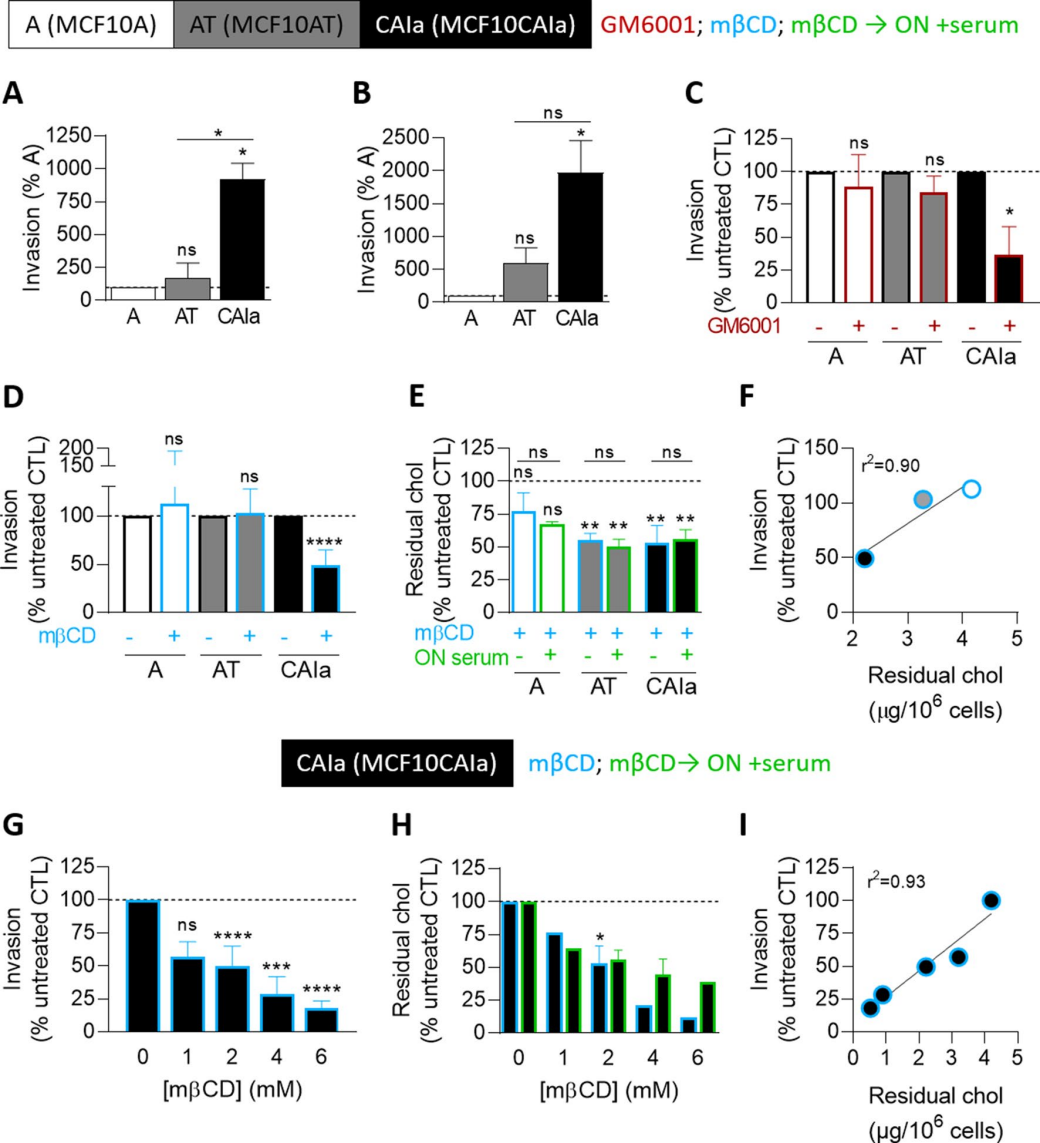
The invasion of the malignant CAIa cells specifically depends on MMP activity and cholesterol content. **A–C** Quantification of invasion of the 3 cell lines in Transwell with a dense Matrigel layer toward 10% serum for 6–12 h (**A** *n* = 5–6 Transwell from 4 independent experiments), 24 h (**B** *n* = 3–4 Transwell from 3 independent experiments) or 6–12 h upon 10 µM GM6001 to impair extracellular matrix degradation (**C** *n* = 4–7 Transwell from 2 to 3 independent experiments). Kruskal–Wallis test followed by Dunn’s comparison test (**A, B**) or Mann–Whitney test (**C**). **D–I** Cell lines were treated with 2 mM (**D–F**) or the indicated concentrations of methyl-β-cyclodextrin (mβCD) for 2 h and then tested for invasion (**D, G**) and residual chol content (**E, H**). Quantification of invasion of the 3 cell lines (**D**) or CAIa (**G**) in Transwell with a dense Matrigel layer toward 10% serum for 6–12 h (*n* = 4–19 Transwell from 2 to 9 independent experiments). Unpaired *t* test (**D**) and Kruskal–Wallis test followed by Dunn’s comparison test (**G**). Residual chol content of the 3 cell lines (**E**) or CAIa (**H**) assessed by Amplex Red directly (blue border columns) or 12 h after mβCD treatment (ON, overnight; green border columns; *n* = 1–5 independent experiments). Kruskal–Wallis test followed by Dunn’s comparison test (**E, H**). Linear correlation between the invasion of the 3 cell lines and their residual chol content (**F**); or between the invasion of CAIa and their residual chol (**I**)

To determine the invasion potential of the three MCF10A cell lines, we used Transwells coated with Matrigel in the presence of 10% serum gradient in the bottom well. Modulation of confinement (through 0.5 mg mL^−1^-loose and 2 mg mL^−1^-dense Matrigel layers) and time of invasion (6, 12 and 24 h) revealed that low confinement favored the pre-malignant AT cell invasion while high confinement was needed to increase malignant CAIa cell invasion, no matter the time of the assay (Fig. S1, Fig. 1A, B). We, therefore, chose a 12 h invasion assay in a dense 2 mg mL^−1^ Matrigel layer to better discriminate the three cell lines for their invasion. Upon global inhibition of extracellular matrix metalloproteinases (MMP) activity with GM6001, the malignant cell invasion was specifically decreased (Fig. 1C).

We then asked whether the differential extent of invasion of the three cell lines could be related to their differential cytocortex and PM stiffness as well as their distinct chol content and surface distribution [23]. Cell invasion seemed to inversely associate with the extent of cytocortex stiffness and total chol content but positively with the extent of PM stiffness and surface chol content (compare Fig. 1A, B with Fig. S2). Since chol is a key regulator of PM properties including stiffness [18, 31, 32], we further investigated the specific dependence of the malignant cells to this lipid for invasion. The membrane chol content was depleted by a 2 h incubation with 2 mM methyl-β-cyclodextrin (mβCD). This treatment was not cytotoxic and was specific towards chol as revealed by thin layer chromatography (Fig. S3). The ~ 30–45% depletion obtained, which was maintained after 12 h incubation in an mβCD-free medium, induced a specific decrease of CAIa cell invasion (Fig. 1D, E). Invasion and residual chol content were very well correlated (Fig. 1F), which was confirmed upon incubation of CAIa cells with increasing concentrations of mβCD (Fig. 1G–I). Altogether, the differential extent of invasion of the pre-malignant vs malignant cells suggested a distinct mode of invasion compatible with the specific dependence of the malignant cells on MMP activity and chol content.

### The surface cholesterol of malignant cells is distributed in submicrometric domains that can be specifically and reversibly decreased by cholesterol depletion

**Fig. 2:**
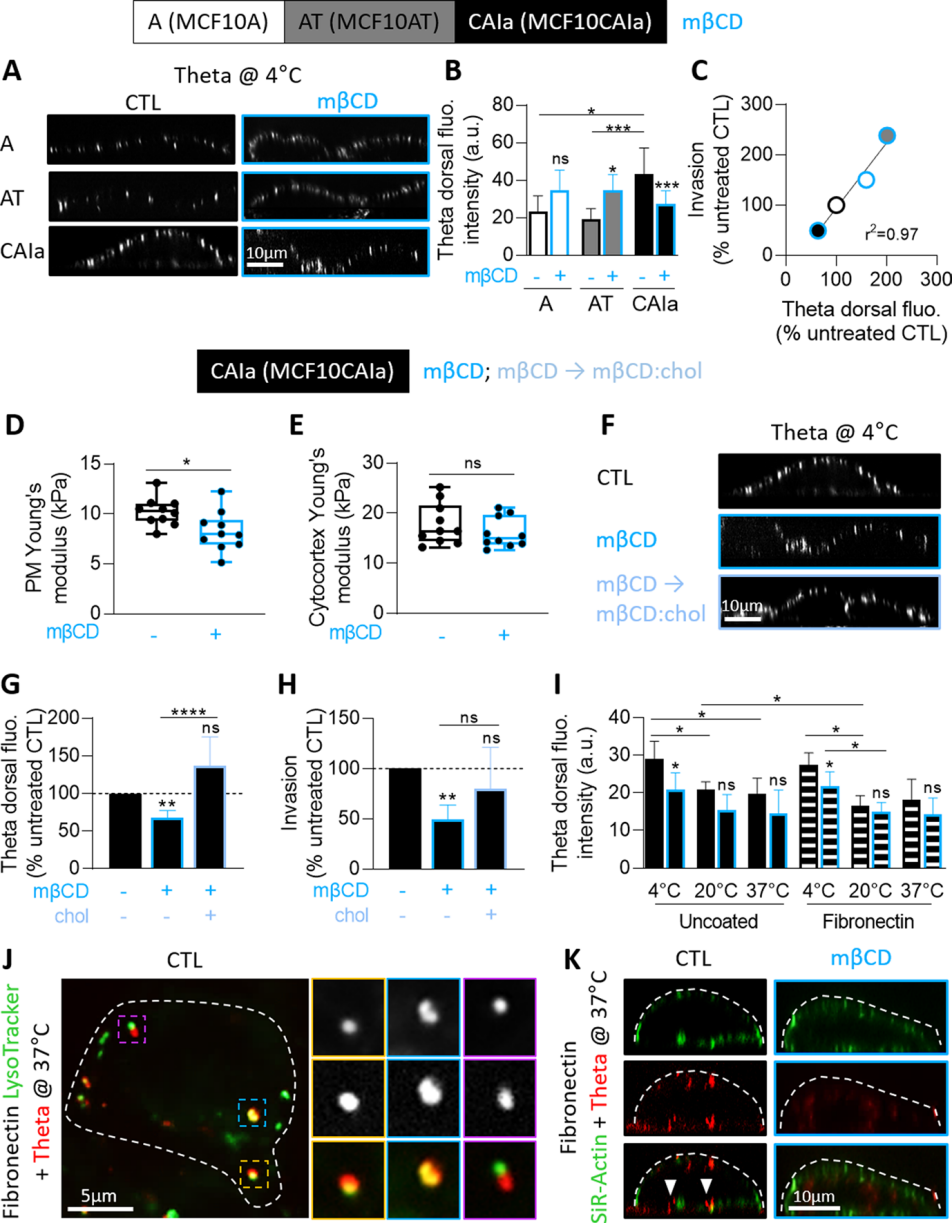
In malignant CAIa cells, the dorsal cholesterol distributes in submicrometric domains that are specifically and reversibly decreased by methyl-β-cyclodextrin and can be endocytosed and reach the ventral face. Cell lines were treated or not (CTL, black) with 2 mM mβCD for 2 h (blue) or repleted with chol after depletion (light blue) and then tested for surface chol content (**A–C, F, G, I, K**), cell stiffness (**D, E**), cell invasion (**H**) and intracellular trafficking (**J**). (**A, F**) X–Z reconstructions of confocal images of the 3 cell lines (**A**) or CAIa (**F**) plated on glass coverslips, treated or not with mβCD followed or not by chol repletion and labeled at 4 °C with the mCherry-Theta toxin fragment specific to endogenous chol. (**B**, **G**) Quantification of the Theta dorsal fluorescence intensity of the 3 cell lines or CAIa treated or not with mβCD followed by chol repletion (average value of 10–15 cells from *n* = 2–28 images from 2 to 8 independent experiments and 15–20 cells from n = 8–14 images from 2 independent experiments). Mann–Whitney test (**B**) and Kruskal–Wallis test followed by Dunn’s comparison test (**B, G**). **C** Linear correlation between mβCD-treated invasion and Theta dorsal fluorescence intensity. (**D**, **E**) CAIa PM and cytocortex Young’s modulus determined by atomic force microscopy. Data points correspond to the mean values measured on a single cell (10 different cells) during *n* ≥ 3 independent experiments. Mann–Whitney test. **H** Quantification of invasion of CAIa in Transwell with a dense Matrigel layer toward 10% serum for 12 h (*n* = 8–9 Transwell from 3 independent experiments). Kruskal–Wallis test followed by Dunn’s comparison test. **I** Quantification of the Theta dorsal fluorescence intensity of CAIa plated on glass (filled bars) or fibronectin-coated coverslips (striped bars), treated or not (black border columns) with mβCD (blue border columns) and labeled at 4, 20 or 37 °C with the mCherry-Theta toxin fragment (10–20 cells from *n* = 3–6 images from 1 experiment). Kruskal–Wallis test followed by Dunn’s comparison test and Mann–Whitney test. **J** Theta intracellular behavior determined by vital imaging at 37 °C on fibronectin-plated CAIa colabeled with the mCherry-Theta toxin fragment and the LysoTracker probe to label late endosomes/lysosomes. The first two insets show LysoTracker-positive compartments containing Theta-labeled chol whereas the third one shows a LysoTracker-negative Theta structure (*n* = 2). **K** X–Z reconstructions of confocal images of CAIa plated on fibronectin-coated coverslips, treated or not with mβCD and colabeled at 37 °C with the mCherry-Theta toxin fragment and the SiR-Actin. Arrowheads, ventral chol-enriched domains (*n* = 1)

To explore the mechanism behind the chol-dependent invasion of the malignant cells, we first evaluated the chol surface distribution by cell labeling at 4 °C with the chol-specific mCherry-Theta toxin which specifically binds to chol and allowed us to reveal chol-enriched domains on living red blood cells ([20]; Fig. S4). The signal at the dorsal surface was then quantified on X–Z reconstructions. The chol surface content was higher in malignant CAIa cells, was distributed into submicrometric domains and was decreased upon mβCD (Fig. 2A, B). Such distribution agreed with our previous data obtained by AFM using Theta toxin derivatized tips which allowed to evidence chol-enriched domains in a label-free manner with high spatial resolution [23]. In addition, it was confirmed by PM insertion of the fluorescent chol analog TopFluor-Chol [33–35]. Thus, X–Z reconstructions and profiles revealed a heterogeneous distribution in clusters similar in size and mβCD sensitivity to those observed upon Theta labeling (Fig. S5A, B). Moreover, TopFluor-Chol well colocalized with mCherry-Theta toxin fragment, whatever the labeling sequence (Fig. S5C, D). In contrast, in normal and pre-malignant cells, domains were less abundant in control conditions but increased after chol depletion (Fig. 2A, B). Combination of data in the three cell lines with cell invasion potential shown in Fig. 1 indicated that the higher the surface chol content, the higher the extent of invasion (Fig. 2C). The specificity of mβCD towards the PM was proved by AFM since a slight decrease of the PM stiffness was observed but the cytocortex stiffness was preserved (Fig. 2D, E). Moreover, the effect of mβCD on cell invasion was reversed by chol repletion (light blue columns, Fig. 2F-H), as an additional argument against mβCD toxicity. In conclusion, the specific role of chol in malignant cell invasion could result from its increased content at the dorsal surface through the formation of submicrometric domains.

### The dorsal surface cholesterol pool removed by methyl-β-cyclodextrin in malignant cells can be internalized by endocytosis and reach the ventral side

To then examine whether chol-enriched domains could also associate with the ventral surface of malignant cells near invadopodia, cells plated on glass or fibronectin-coated coverslips were compared for chol distribution at the dorsal side after labeling at 4, 20 and 37 °C. Fibronectin coating did not significantly affect the dorsal surface chol content as compared to glass coverslips, except for a slight decrease at 20 °C (Fig. S6 and filled columns vs striped columns in Fig. 2I). In contrast, increasing the temperature significantly decreased the dorsal chol content at the benefit of intracellular and ventral labeling (Fig. 2I and red vs yellow arrowheads in Fig. S6), suggesting endocytosis of the Theta toxin at 20 and 37 °C. This was confirmed by the observation that the majority of Theta-positive structures colocalized with LysoTracker in living CAIa cells colabeled with the Theta toxin and the LysoTracker to evidence acidic endocytic compartments (Fig. 2J idem and S7A). Moreover, inhibition of endocytosis by the dynamin inhibitor Dyngo4a reversibly abrogated the redistribution of Theta clusters at the ventral side of CAIa cells (Fig. S7B, C). In addition, the mβCD-induced decrease of chol exposure previously observed at 4 °C was abrogated at 20 or 37 °C (Fig. S6 and blue border columns in Fig. 2I), suggesting that the chol pool that can be extracted by mβCD can reach the ventral face. This was confirmed by co-labeling CAIa cells with the Theta toxin and the SiR-actin probe. X–Z reconstructions showed the presence of chol at the ventral face in proximity with actin structures in untreated cells contrasting with its absence in mβCD-treated cells (Fig. 2K). Those observations suggested that the surface chol pool removed by mβCD can be internalized and reach the cell ventral face.

### Partial membrane cholesterol removal in malignant cells does not impair the distribution of microtubules, intermediate filaments and focal adhesions but decreases invadopodia size and abundance

**Fig. 3:**
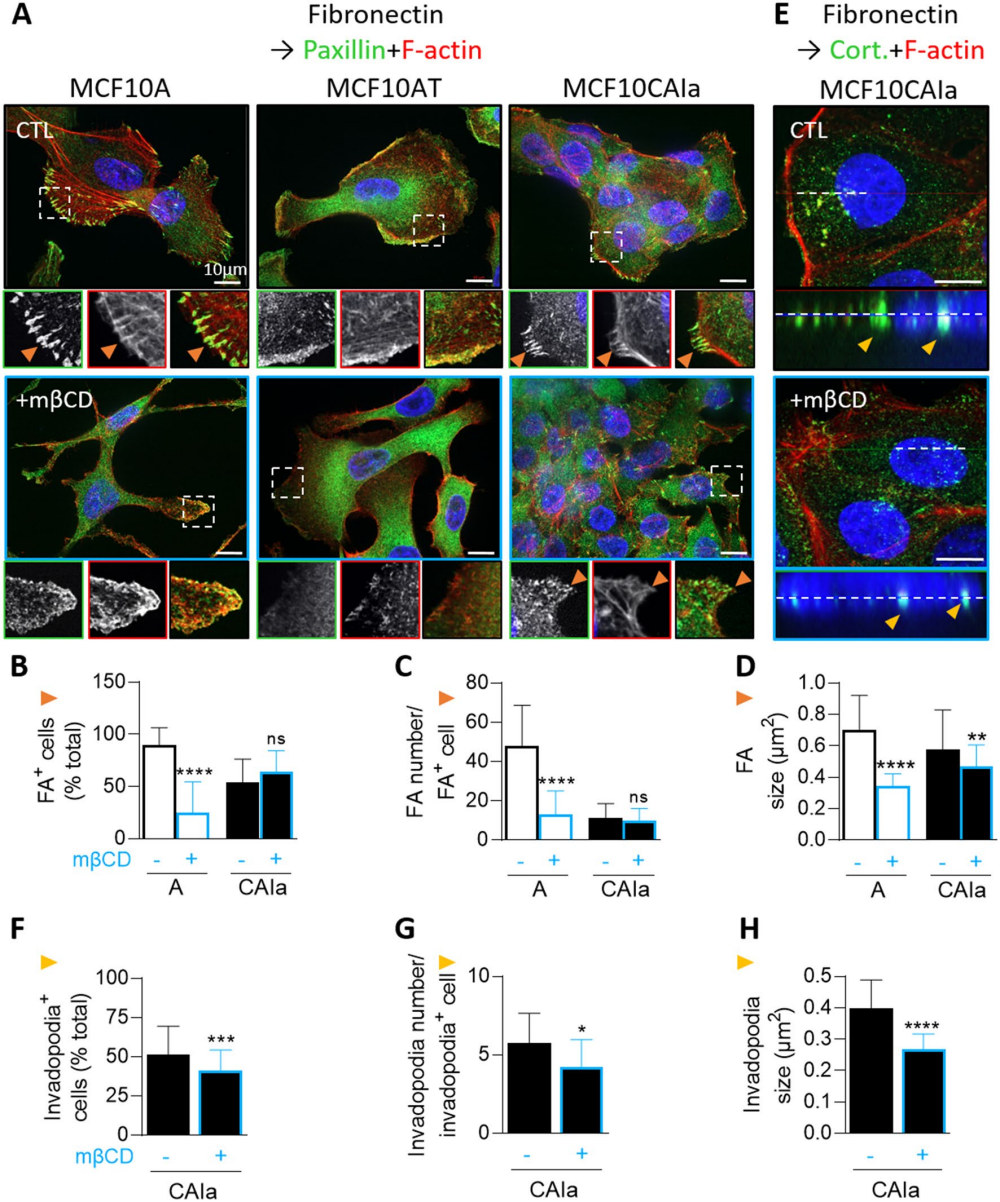
Cholesterol depletion in malignant CAIa cells does not affect focal adhesions but reduces invadopodia size and abundance. Cell lines were plated on fibronectin-coated coverslips, treated with 2 mM mβCD for 2 h (blue), immunolabeled for paxillin (**A–D**) or cortactin (**E–H**), analyzed by confocal microscopy and quantified. **A**–**E** Confocal images of the 3 cell lines (**A**) or CAIa (**E**) immunolabeled with anti-Paxillin or anti-Cortactin together with F-actin (stained by Phalloidin) and nuclei (stained with Hoechst). Insets in **A** and orange arrowheads show paxillin-positive focal adhesions (FA). Insets in **E** and yellow arrowheads show X–Z reconstructions of invadopodia length along the dotted line. **B**–**D** Quantifications of the number of A and CAIa cells presenting focal adhesions, the number of focal adhesions per cell and the focal adhesion size [*n* = 27–35 images (**B**) and *n* = 44–84 cells (**C, D**) from 4–5 independent experiments]. **F–H** Quantifications of the number of CAIa cells presenting invadopodia, the number of invadopodia per cell and the invadopodia size (*n* = 16–65 images from 2 independent experiments). Unpaired *t* test

As malignant CAIa cells exhibit the lowest cytocortex stiffness and the highest chol surface content and invasion potential (Figs. 1B, 2B, S2A, D), we compared the cytoskeleton organization of the three cell lines and their interplay with chol. Immunolabeling of α-tubulin showed a similar spread pattern in the three cell lines that was not modified by chol depletion (Fig. S8A, S9A). Intermediate filament organization was differential in the three cell lines, with vimentin clustering around the nuclei in pre-malignant and malignant cells *vs* higher spreading in A cells, but also not affected by chol depletion (Fig. S9B). This differential pattern was accompanied by a distinct extent of vimentin dimerization (Fig. S8B, C). Focal adhesions revealed by paxillin immunolabeling were also differentially organized, with a lower number in malignant cells as compared to normal ones. Moreover, chol depletion strongly impaired the formation of focal adhesions in the normal cells but not in the malignant ones (Figs. 3A–D, S8D, S10A). Finally, invadopodia, which were revealed in CAIa cells by serum starvation followed by stimulation with serum-containing medium and immunolabeling for cortactin (Figs. S8E, S10B; [36]), were decreased in abundance and size upon chol depletion (Fig. 3E–H). In conclusion, chol depletion in malignant CAIa cells did not impair the distribution of microtubules, intermediate filaments and focal adhesions but decreased invadopodia size and abundance, suggesting that membrane chol could be involved in the formation of right-sized invadopodia.

### Partial membrane cholesterol removal in malignant cells reversibly decreases extracellular matrix degradation

**Fig. 4:**
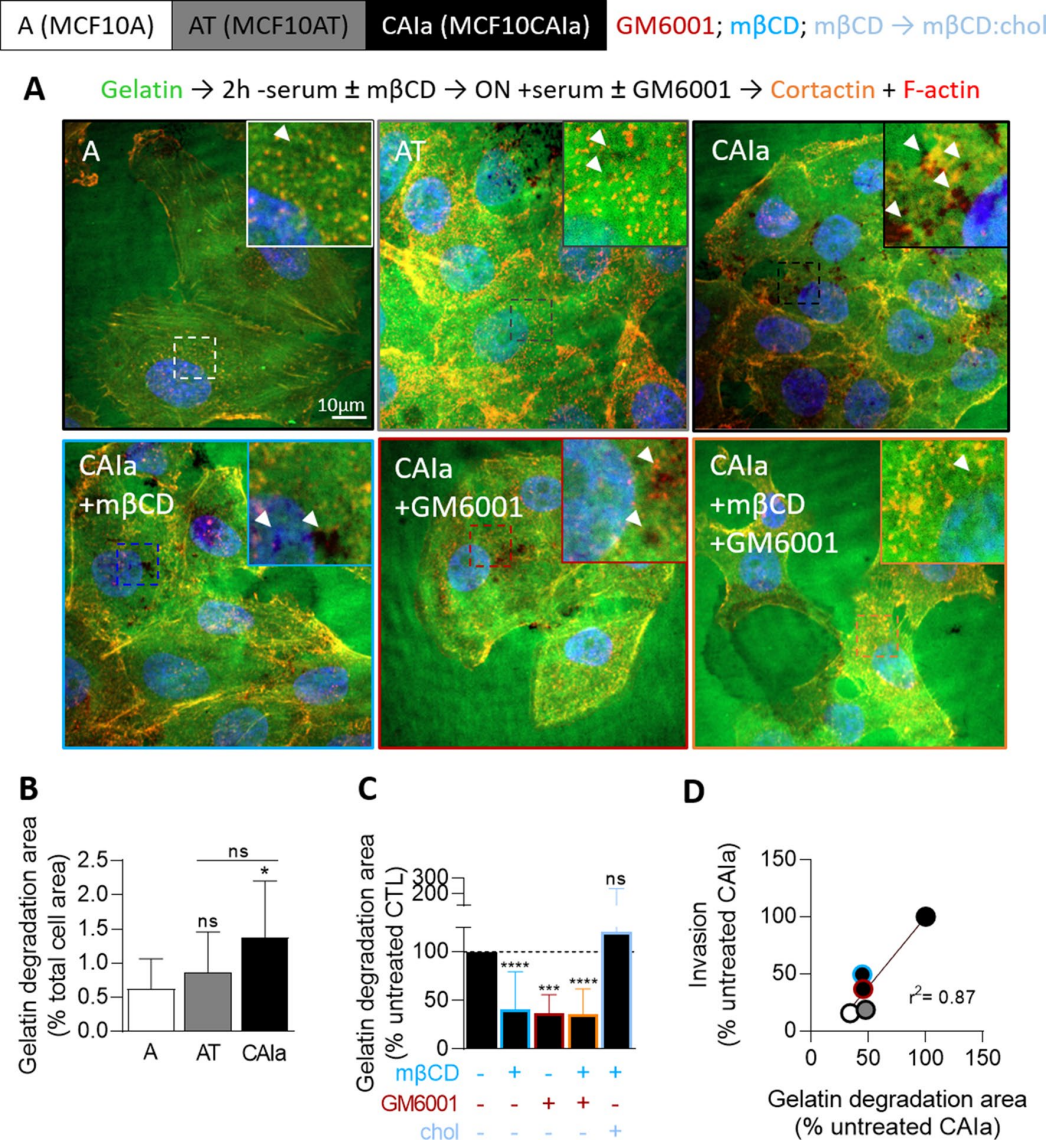
Cholesterol depletion in malignant CAIa cells reduces gelatin degradation to a similar extent than MMP inhibition. Cell lines were plated on Oregon Green-coated coverslips, serum-starved combined or not with 2 mM mβCD (blue) for 2 h, then repleted or not with chol for 1 h (light blue). Cells were then stimulated overnight (ON) with serum-containing medium supplemented or not with GM6001 (red) and then tested for gelatin degradation. **A** Representative images of gelatin degradation (black areas; arrowheads) of cells (immuno)labeled with anti-Cortactin, F-actin (Phalloidin) and nuclei (Hoechst). **B**, **C** Quantification of gelatin degradation potential of the 3 cell lines (**B** 5–20 cells from *n* = 8–12 images from 1 experiment) or CAIa treated or not with mβCD, GM6001 or the combination of both drugs or repleted with chol (**C** 10–30 cells from *n* = 9–50 images from 1 to 4 independent experiments). Kruskal–Wallis test followed by Dunn’s comparison test. **D** Linear correlation between the invasion of the 3 cell lines and CAIa treated with mβCD in combination or not with GM6001 and gelatin degradation potential

We next examined the consequences of mβCD-induced impaired invadopodia on ECM degradation using a gelatin degradation assay in cells plated on Oregon Green-coated coverslips, using the protocol of serum starvation and immunolabeling described above. Malignant CAIa cells exhibited the highest gelatin degradation potential (Fig. 4A, B). Chol depletion in CAIa cells significantly decreased gelatin degradation in a reversible manner and similarly to MMP inhibition by GM6001 (Fig. 4C). Combination of mβCD and GM6001 treatments did not induce a stronger effect and the level of gelatin degradation potential of the three cell lines, untreated or not, positively correlated with the invasion potential (Fig. 4D). All those data suggested that mβCD and GM6001 could have the same target, potentially invadopodia sites.

### The effects of cholesterol depletion on cell invasion, cholesterol surface exposure and invadopodia size in malignant CAIa cells are abrogated upon actin polymerization inhibition

**Fig. 5:**
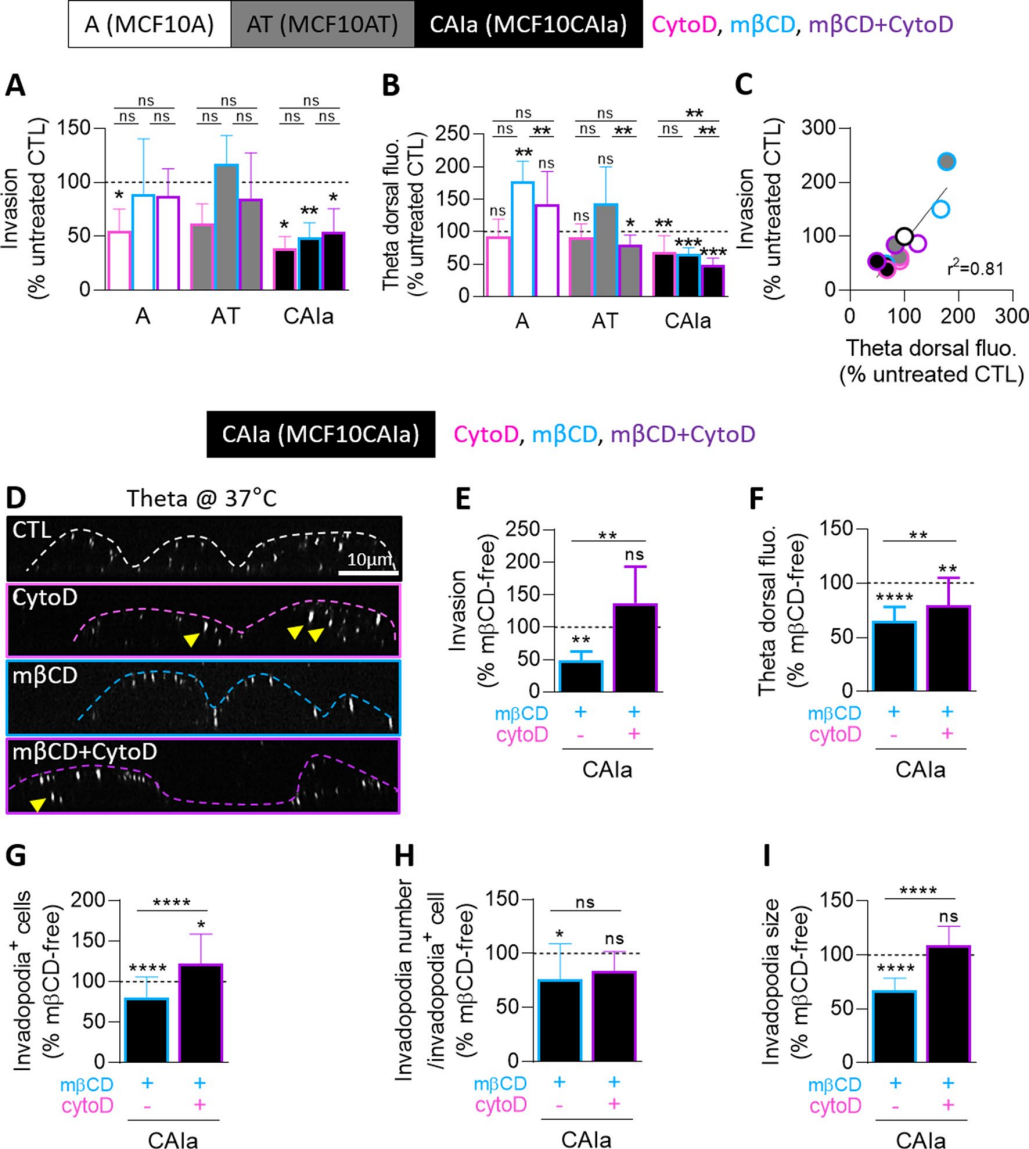
Actin polymerization inhibition in malignant CAIa cells decreases the effect of cholesterol depletion on invasion, cholesterol surface exposure and invadopodia size. Cell lines were treated for 2 h with 2 mM mβCD (blue) in combination (purple) or not with 0.5 µM cytochalasinD (cytoD; pink) and then tested for invasion, surface chol content and invadopodia. **A**, **E** Quantification of invasion of the 3 cell lines (**A**) or CAIa (**E**) in Transwell with a dense Matrigel layer toward 10% serum for 6–12 h (*n* = 5–9 Transwell from 2–3 independent experiments). **B**, **F** Quantification of the Theta dorsal fluorescence intensity of the 3 cell lines (**B**) or CAIa (**F**; 5–15 cells from *n* = 7–21 images from 2 to 6 independent experiments). Data in E,F are expressed in the percentage of mβCD-free conditions. Kruskal–Wallis test followed by Dunn’s comparison test (**A, B**) and Mann–Whitney test (**E, F**) to compare all treatments per cell line and Wilcoxon signed-rank test to compare treatments with untreated CTL (**A, B, E, F**). **C** Linear correlation between cell invasion and Theta dorsal fluorescence intensity. **D** X–Z reconstructions of confocal images of CAIa plated on fibronectin-coated coverslips, treated or not with mβCD in combination with cytoD and then labeled at 37 °C with the mCherry-Theta toxin fragment. Arrowheads, intracellular chol sequestration. **G**–**I** Quantification of the number of cells presenting invadopodia, the number of invadopodia per cell and the invadopodia size of CAIa plated on fibronectin-coated coverslips, treated with mβCD in combination or not with cytoD and immunolabeled with anti-Cortactin (*n* = 8–50 images from 2 independent experiments). One sample *t* test and unpaired *t* test (**G**) and Wilcoxon signed-rank test and Mann–Whitney test (**H, I**)

As chol could be found in the proximity of ventral actin structures (Fig. 2K) and since chol controlled invadopodia size (Fig. 3E–H), we then tested if the actin cytoskeleton could in return control dorsal chol distribution. To test this hypothesis, cells were pretreated with mβCD, cytochalasin D (cytoD, an inhibitor of actin polymerization) or the combination of both drugs and assessed for invasion and for chol, focal adhesion and invadopodia distribution. CytoD disturbed F-actin organization particularly on the ventral side of malignant CAIa cells, decreased the size and abundance of focal adhesions in A and CAIa cells but did not significantly impact invadopodia in CAIa cells (pink at Fig. S11). Upon cytoD, invasion of the three cell lines was reduced but more strongly in CAIa cells and the surface chol content was only significantly decreased in CAIa cells (Fig. 5A, B). The latter effect could result from the intracellular chol sequestration evidenced by Theta labeling at 37 °C (pink in Fig. 5D), in agreement with [37]. Upon cytoD combined with mβCD, cell invasion and chol surface distribution were also affected in the same way (mauve at Fig. 5A, B), as confirmed by the strong and positive correlation between those two parameters (Fig. 5C). More importantly, inhibition of actin polymerization in the malignant cells decreased or even abrogated the mβCD-induced impaired invasion, chol surface exposure and invadopodia abundance and size (Fig. 5E-I, S12), suggesting an interplay between F-actin and chol functionality in the malignant cells.

### The ER spreading and ER-organelle contact sites are more extended in malignant cells and perturbed by cholesterol depletion

**Fig. 6:**
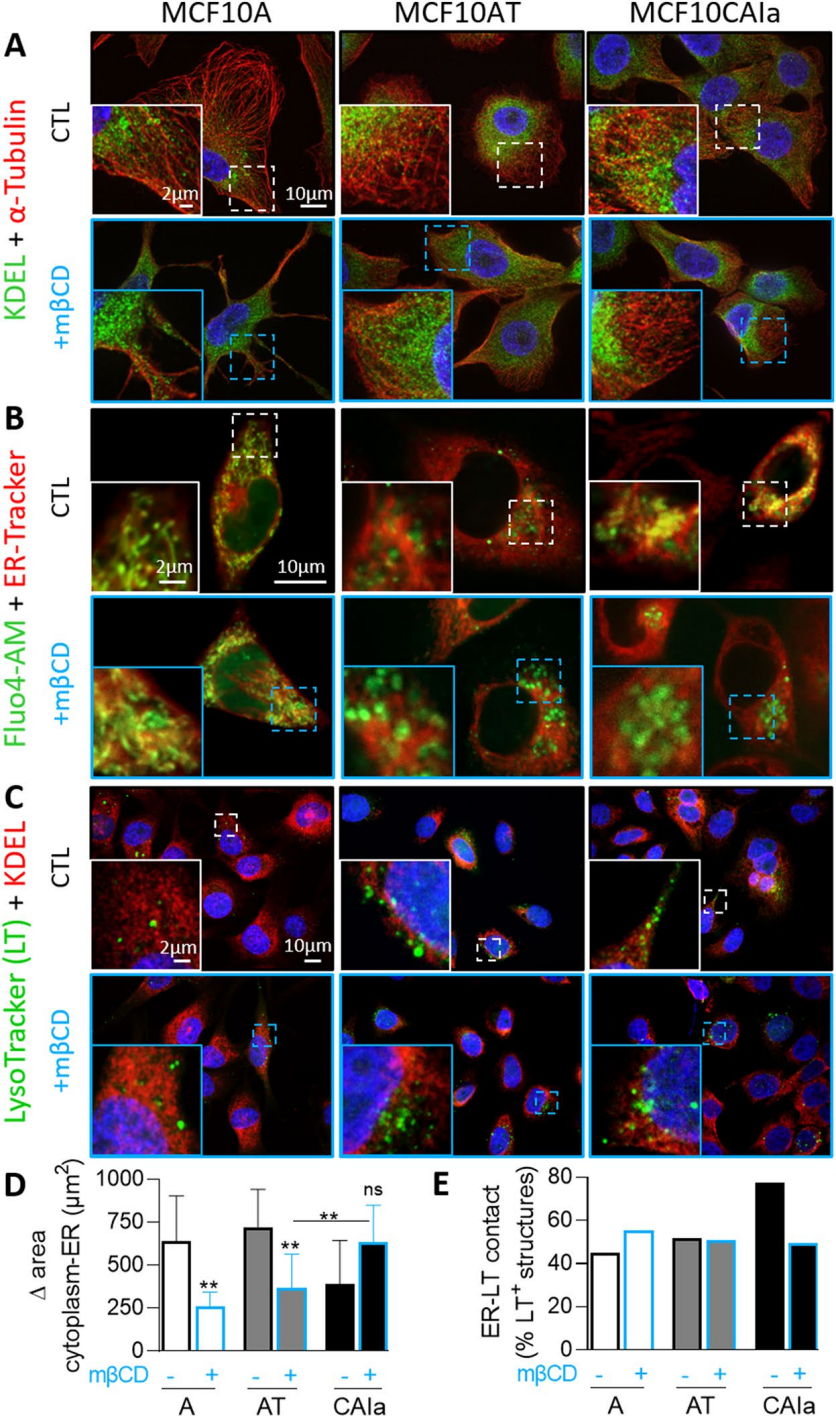
The higher endoplasmic reticulum network spreading and its closer contact with mitochondria/lysosomes in malignant cells than in normal and premalignant cells are lost upon cholesterol depletion. The 3 cell lines were plated on fibronectin-coated coverslips, treated or not with 2 mM mβCD for 2 h (blue) and analyzed by confocal microscopy. **A** Coimmunolabeling with anti-KDEL (endoplasmic reticulum; ER) and anti-α-Tubulin (microtubules, to reveal the whole cytoplasm). Nuclei stained with Hoechst. **B** Colabeling at 37 °C with the Fluo4-AM specific to Ca^2+^ and the ER-Tracker. **C** labeling at 37°C with the LysoTracker (LT) specific to late endosomes/lysosomes followed by immunolabeling with anti-KDEL. **D** Quantification of the ER network spreading of the 3 cell lines treated or not (black border columns) with 2 mM mβCD for 2 h (blue border columns). Data are expressed as the difference of area between the whole cytoplasm and the ER (*n* = 12–47 cells from *n* = 7–8 images from 1 experiment). Kruskal–Wallis test followed by Dunn’s comparison test and Mann–Whitney test. **E** Quantification of the number of LysoTracker (LT) positive structures in close contact with the ER in the 3 cell lines treated or not (black border columns) with 2 mM mβCD for 2 h (blue border columns). Data are expressed as ER-LysoTracker (LT) contact structures reported to total LysoTracker positive structures (*n* = 29–50 profiles from 8 to 14 images from 1 experiment)

As the mechanism behind the higher distribution of chol at the surface of CAIa cells, we hypothesized a differential organization of endoplasmic reticulum (ER)-PM contact sites which are involved in the regulation of lipid transport a.o.. The three cell lines were thus immunolabeled for the ER marker KDEL and α-tubulin or cortactin to, respectively, reveal the whole cell or the ventral side (Fig. 6A, S13A). Whereas the ER was spread throughout the cytoplasm in malignant CAIa cells, it was more restricted to the perinuclear region in A and AT cells. Upon mβCD, the opposite situation was seen with a higher spreading in A and AT cells as compared to the CAIa cells (Fig. 6A, D).

To then explore whether the differential ER spreading in the three cell lines could be accompanied by differential ER-mitochondria contact sites, we analyzed the distribution of the ER *vs* Ca^2+^ ions in living cells using an ER-Tracker and Fluo4-AM. Mitochondria are indeed associated with Ca^2+^ handling [38]. The Fluo4-positive structures exhibited a tubular structure typical of mitochondria in normal cells but a more rounded morphology in pre-malignant and malignant cells, in agreement with [39]. Moreover, the contact/colocalization between those Ca^2+^-enriched structures and the ER was more visible in malignant cells than in normal and pre-malignant ones and was decreased by chol depletion (Fig. 6B). We finally explored the ER-endocytic compartment contact sites by KDEL immunolabeling in Lysotracker-labeled cells. The Lysotracker-positive structures were less abundant (Fig. S13B) and exhibited a wider and more aligned distribution and closer contacts with the ER in malignant cells than A and AT cells. All those particularities were abrogated by mβCD (Figs. 6C, E, S13C). Altogether, those data suggested that the extent of ER-PM and ER-organelle contact sites were differentially regulated in malignant cells and were dependent on the chol content.

### MDA-MB-231 cells also depend on cholesterol for invasion, invadopodia size and gelatin degradation

**Fig. 7:**
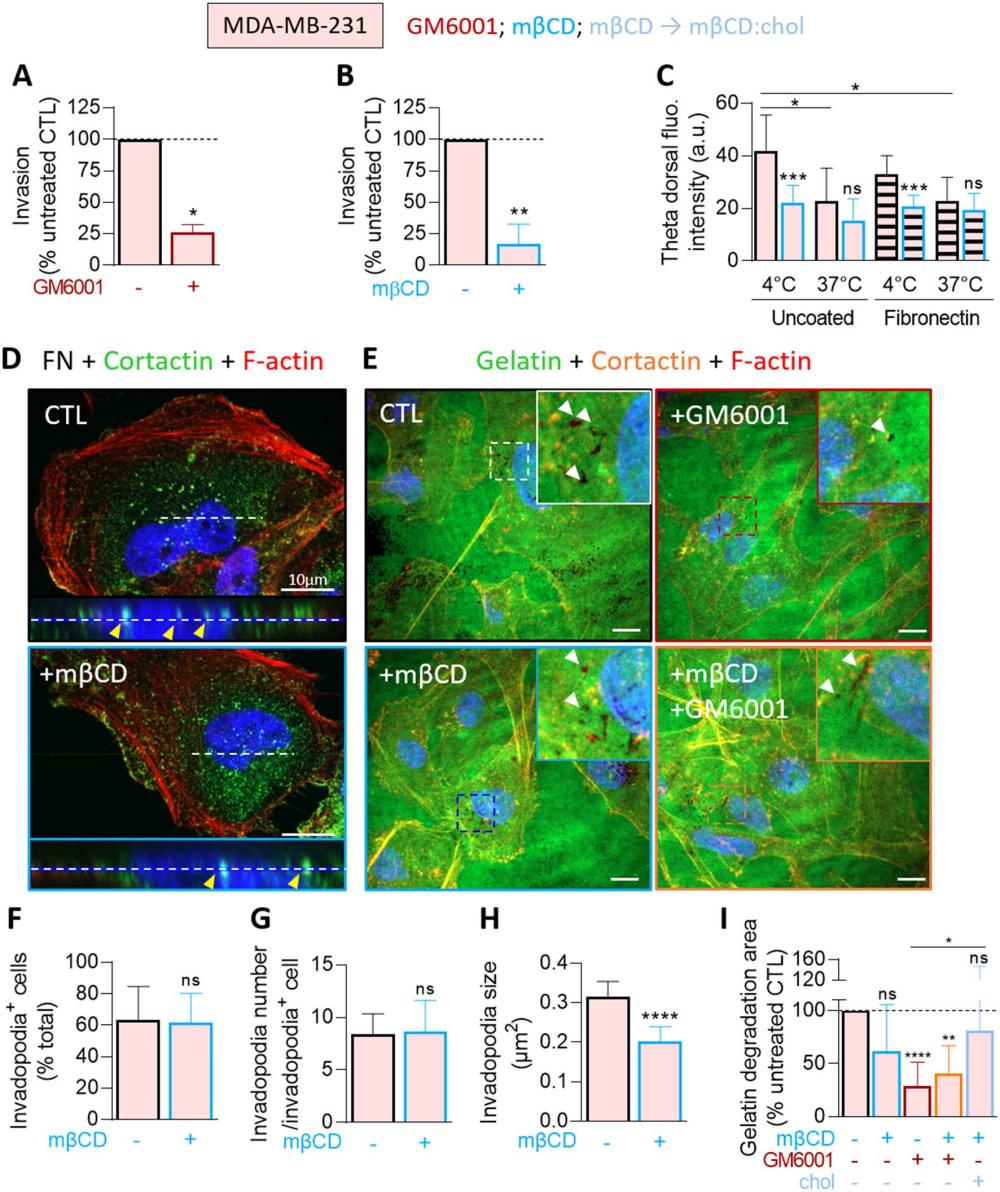
Malignant MDA-MB-231 cells also present high surface cholesterol distribution and depend on cholesterol for invasion, invadopodia size and gelatin degradation. MDA-MB-231 were treated with 2 mM of mβCD (blue) in combination or not with 10 µM GM6001 (red) or repleted with chol for 1 h (light blue) and then tested for invasion (**A, B**), surface chol content (**C**), invadopodia formation (**D, F–H**) and gelatin degradation (**E, I**). **A**, **B** Quantification of invasion in Transwell with a dense Matrigel layer toward 10% serum for 12 h upon GM6001 or after 2 h of mβCD (*n* = 4–5 Transwell from 2 independent experiments and *n* = 6 from 3 independent experiments, respectively). Mann–Whitney test. **C** Quantification of Theta dorsal fluorescence intensity of MDA-MB-231 plated on glass (filled bars) or fibronectin-coated coverslips (striped bars), treated or not (black border columns) with mβCD (blue border columns) and labeled at 4 or 37 °C with the mCherry-Theta toxin fragment (7–15 cells from *n* = 8–11 images from 2 independent experiments). Mann–Whitney test. **D**, **E** Confocal images of MDA-MB-231 plated on fibronectin (FN)-coated or Oregon Green-coated coverslips, treated as described in Figs. 3E and 4 and immunolabeled with anti-Cortactin together with F-Actin (Phalloidin) and nuclei (Hoechst). Insets in **D** show X–Z reconstructions of invadopodia length along the dotted line. **F–H** Quantification of the number of cells presenting invadopodia, the number of invadopodia per cell and the invadopodia size from images presented in **D** (*n* = 12–35 images from 2 independent experiments). Unpaired *t* test. **I** Quantification of gelatin degradation potential from images presented in **E** (8–15 cells from *n* = 10–46 images from 1 to 4 independent experiments). One-Way ANOVA test followed by Dunnett’s comparison test

To finally explore the relevance of our data for a more common mutation in breast cancer, analyses were extended to the highly invasive triple-negative MDA-MB-231 cell line. Like malignant CAIa cells, MDA-MB-231 cells exhibited a high invasion potential that depended on both MMP activity and chol content (Fig. 7A, B). Theta labeling on both glass and fibronectin-coated coverslips revealed a similar dorsal chol distribution to CAIa cells. Moreover, this dorsal chol was decreased by temperature increase and chol depletion at 4 °C but not at 37 °C (Fig. 7C). Although the abundance of invadopodia was not impacted by chol depletion, their size was decreased (Fig. 7D, F–H). The ECM degradation was also decreased in a reversible manner and a combination of mβCD and GM6001 treatments did not induce a stronger effect than GM6001 alone, as in CAIa cells (Fig. 7E, I). Altogether, the importance of membrane chol for malignant cell invasion through matrix degradation and right-sized invadopodia formation was confirmed in another invasive breast cancer cell line, the MDA-MB-231 cells.

## Discussion

### Main observations

Using the MCF10A cell line series as a model of breast cancer progression, we have previously reported higher PM stiffness and surface chol content of malignant CAIa cells compared to normal A and pre-malignant AT cells [23]. We here showed that the malignant CAIa cells specifically exhibited submicrometric chol-enriched domains at their surface and chol-dependent narrow contact sites between the ER and PM or organelles. This specific organization was required for the control of cell invasion. Mechanistically, chol-enriched submicrometric domains reached the cell ventral face where they were involved in invadopodia maturation and ECM degradation. Chol surface distribution and chol-dependent control of invadopodia and invasion were similarly observed in MDA-MB-231 cells but contrasted with the lower surface chol exposure and the chol-dependent control of focal adhesions in non-malignant cells.

### Cholesterol forms two types of submicrometric domains at the surface of malignant cell lines

Conflicting data regarding the deregulation of chol in cancer arise in the literature. On one hand, cancer cells exhibit a reprogrammed chol metabolism notably due to enhanced chol biosynthesis [17, 40]. On the other hand, lower chol levels in the human liver and breast cancer cell lines lead to increased membrane fluidity and promote migration and invasion [41–43]. By directly comparing cell lines with the same genetic background, we showed that despite a similar chol content in malignant *vs* non-malignant cells, malignant cells exhibited a ~ 50% increase of chol at the external PM leaflet and its clustering in submicrometric domains.

Due to the high threshold required for chol Theta toxin binding (∼ 30 mol%), domains should present a very high chol content [20]. Domain abundance is decreased by ~ 35% with 2 mM mβCD, in the same order of magnitude than the ~ 45% reduction of the total chol content. Based on those data, our previous data on living red blood cells and myoblasts [20] and the work of Das and coll. on fibroblasts [44], we suggested that two types of chol-enriched domains coexist at the malignant cell surface. The first one is sensitive to mβCD and corresponds to the labile pool described by Das et al., while the second one cannot be removed by mβCD and represents the non-labile pool. This former pool can be internalized, as revealed by the abrogation of mβCD effect on chol-enriched domain redistribution from the dorsal to the ventral face upon temperature increase. Moreover, the chol content of those domains appeared to depend on the actin cytoskeleton, since actin polymerization inhibition by cytoD removed ∼35% of the PM chol specifically in malignant cells and without potentiating the effect of mβCD. The interplay between chol and the actin cytoskeleton has been recently suggested. For instance, disruption of the actin cytoskeleton leads to the movement of sterols from the PM to endosomes [37]. Moreover, the knockout of ORP2, an oxysterol binding protein (OSBP)-related protein identified as a unique transporter of the labile chol pool from the ER to the PM [45], impairs hepatoma cell migration and adhesion through disorganization of F-actin and lamellipodia [46].

### Cholesterol surface exposure in malignant cells associates with narrow membrane contact sites

Converging evidences in the literature indicate that, after synthesis in the ER, the transport of chol is fast and carried between membranes by lipid transfer proteins. We here showed that the ER-PM contact sites were more extended in the malignant cells, in agreement with their proposed role in cancer progression [47, 48]. Moreover, chol depletion decreased both ER-PM contacts and chol surface exposure, consistent with the accumulation of ER-resident proteins at ER-PM contact sites upon chol supplementation [49, 50].

Those data led us to suggest that the increased surface chol content in malignant cells could result from an increase in chol biosynthesis in the ER, followed by exchanges at the ER-PM contact sites. Nevertheless, the total chol content was not increased in malignant cells and ER-anchored lipid transfer proteins should sense and transport accessible PM chol to the ER to maintain lipid homeostasis. Therefore, the mechanism behind the higher cell surface chol exposure should be far from more complex, involving chol PM retention through two non-mutually exclusive potential scenarios.

The first scenario implies chol PM-to-endosome retrograde trafficking and its return to the PM by a vesicular trafficking-independent mechanism. This sterol flow, which has been recently identified in yeast, involves the ER [37]. Accordingly, ER-endosome contact sites are implied in chol exchange through ORP1L, another member of the OSBP family [51]. Three pieces of data specifically observed in malignant cells supported this hypothesis, *i.e.* the extended ER spreading, the chol-dependent ER-PM and ER-endosomes contacts and the partial redistribution of chol from the cell surface to intracellular structures upon actin polymerization impairment.

The second scenario proposes that, after the exchange at ER-PM contact sites, chol is rapidly transferred to the outer PM leaflet, preserving it from retrograde transport from the PM inner leaflet to the ER. Although the transversal PM distribution of chol is far from well understood, sterols have been proposed to partition in the outer leaflet according to their affinity for sphingolipids or in the inner leaflet driven by positive interactions with PE or PS [52]. The fact that PE is less abundant in invasive MCF10CAIa cells compared to premalignant cells [15] and that the cytocortex stiffness is lower in malignant cells compared to pre-malignant and normal ones [23] supported the possibility of a lower chol retention in the inner PM.

### Cholesterol surface exposure in malignant cells specifically controls invasion

The invasion potential of the two invasive cell lines, the CAIa and MDA-MB-231, was specifically and reversibly inhibited by chol depletion. The contribution of chol in cell migration/invasion is not new but data are often contradictory. Indeed, chol depletion has been shown to reduce breast cancer cell lines migration [53, 54] while its supplementation promotes migration and invasion of renal carcinoma cells [55]. Conversely, lower membrane chol content correlates with increased invading capacity compared to non-invading cells [13, 19].

**Fig. 8:**
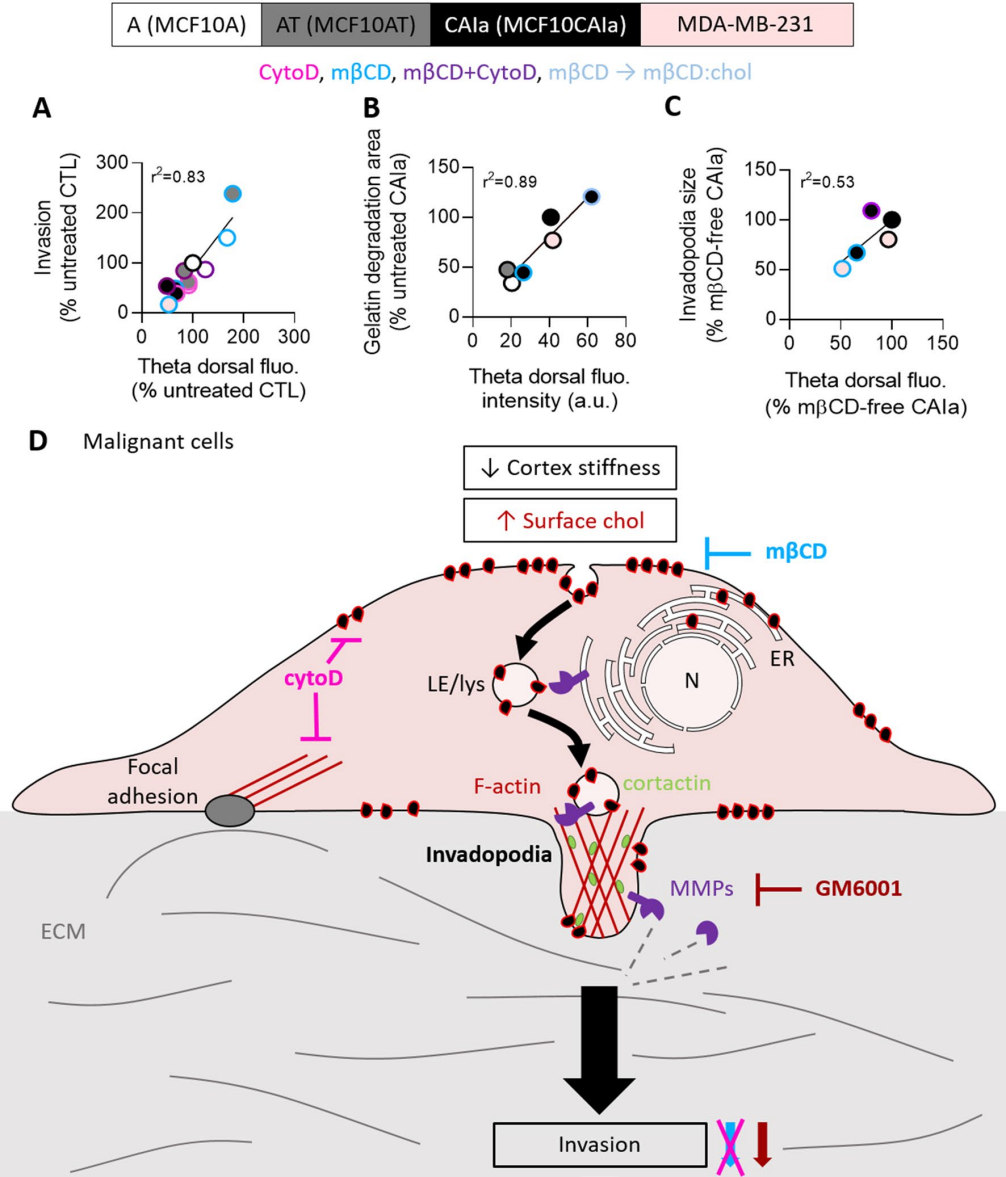
Hypothetical model. Positive linear correlations between the Theta dorsal fluorescence intensity and cell invasion (**A**), gelatin degradation potential (**B**) and invadopodia size (**C**) in MCF10A (white), AT (grey), CAIa (black) and MDA-MB-231 (light pink) treated with mβCD (blue border) in combination (purple border) or not with cytoD (pink border) or repleted with chol (light blue border). **D** Hypothetical model for the implication of membrane chol-enriched domains in malignant cell invasion through their endocytosis from the dorsal side, exchange of MMPs at ER-late endosome/lysosome contact sites and transport into invadopodia. In addition, dorsal and ventral chol-enriched domains could also contribute to invadopodia signaling events (not shown). For additional information, see discussion. N, nucleus; ECM, extracellular matrix; ER, endoplasmic reticulum; LE, late endosome; Lys, lysosome. Thick arrows, pathway favored in malignant *vs* non-malignant cells

In addition, the specificity towards malignant *vs* non-malignant cells and the mechanisms behind the implication of chol are far from well understood. We here proved the specific role of chol in the invasion of malignant cells through the comparison with normal and pre-malignant ones. Mechanistically, we evidenced that chol-enriched surface domains played a key role in breast cancer cell invasion, as supported by the following lines of evidence. First, through the use of mβCD combined with imaging with validated chol probes, we showed that the high chol surface and invasion extent were similarly impaired by chol depletion, specifically in malignant cells. Second, the effect of mβCD on cell invasion was abrogated in cytoD-treated malignant cells and accompanied by a reduced chol surface exposure. Thus, we revealed the implication of chol surface content in cell invasion, a conclusion supported by the excellent correlation between those two parameters, whatever the cell line and the pharmacological treatment (Fig. 8A).

### Cholesterol surface exposure in malignant cells controls invadopodia maturation and ECM degradation

Previous studies have shown that PM chol appropriate levels in human melanoma cells and caveolin-1 in breast cancer cells regulate the formation and function of invadopodia [56, 57] and that disruption of lipid rafts by mβCD suppresses invadopodia formation and breast cancer cell invasion [58]. Nevertheless, those conclusions were mostly based on indirect evidences. Combining the Theta toxin fragment at different temperatures to label both the surface and intracellular chol pools with chol modulation approaches, we here showed that the high chol surface exposure in malignant cells contributed to control invasion through right-sized invadopodia and ECM degradation. Invadopodia formation requires three phases. The initiation phase is characterized by the assembly of actin-based precursor complexes and cortactin-dependent actin polymerization that extends the PM and drives elongation of cellular protrusions and is facilitated by microtubules and intermediate filaments. During the stabilization phase, actin filaments are crosslinked into tightly packed bundles and anchored to PM to form a stable 3D functional structure. During the maturation phase, MMPs are recruited to invadopodia, allowing for local ECM degradation. Our data indicated that chol depletion did affect neither microtubules, intermediate filaments and invadopodia abundance nor ECM degradation area formation but decreased the size of both invadopodia and ECM degradation area in correlation with the residual chol content (Fig. 8B, C). This suggested no effect on the initiation phase but impairment of invadopodia maturation, a step known to depend on anterograde transport of MT1-MMP [36]. This proteolytic enzyme is, like PM chol, endocytosed by clathrin- and caveolin-dependent pathway [59], depends on the aggregation of F-actin and cortactin to accumulate at invadopodia [60], associates with lipid rafts [57] and depends on membrane contacts with the ER-resident protein protrudin to mediate ECM degradation [36]. In support of the above hypothesis, we found that dorsal chol-enriched domains can reach the malignant cell ventral side by endocytosis and that contact sites between the ER and late endosomes/lysosomes in malignant cells were closer and chol-dependent. We thus suggested that chol-enriched domains contribute to malignant cell invasion through their endocytosis from the cell dorsal side, exchange of MMPs at ER-late endosome/lysosome contact sites and transport at the ventral side into invadopodia (Fig. 8D).

As a non-mutually exclusive mechanism, chol-enriched domains could contribute to signaling events at the different stages of invadopodia formation and maturation which are under distinct but interconnected signaling control mechanisms. For instance, growth factor signaling promotes the formation of invadopodia, whereas adhesion to the ECM promotes localized protease exocytosis [61]. Several studies support the role of chol-enriched domains in growth factor receptor distribution, endocytosis and signaling at the PM. Thus, using single-molecule optical tracking, Lin and coll. have revealed chol-mediated interactions between activated EGFRs at the surface of living cells [62]. In prostate tumor cells, the EGFR is phosphorylated and more active within membrane rafts [63]. In live CHO-K1 PM, part of EGFR in the resting state is trapped in chol-containing domains and after disruption of those domains, EGF induces microscopic EGFR clusters and endocytosis is inhibited [64]. In ovarian carcinoma cells in 3D collagen, EGFR and MT1-MMP colocalize with caveolae prior to activation and stimulation with EGF disrupts their association and leads to MT1-MMP internalization [65]. In addition, MT1-MMP associates with lipid rafts [57], can activate the pro-MMP-2 through the formation of a complex with the pro-MMP2 and the tissue inhibitor of metalloproteinases-2 TIMP-2 and can process cell adhesion molecules [66].

### Cholesterol in non-invasive cells controls focal adhesions

The high surface chol, the chol- and MMP-dependent invasion and the narrow chol-dependent ER-organelle/PM contact sites in malignant cells contrasted with the lower surface chol exposure, the MMP- and chol-independent invasion and the looser membrane contact sites in non-invasive cells. Moreover, in contrast to its effect in malignant cells, chol depletion increased both the ER spreading and the chol surface exposure in non-malignant cells. These findings agreed with the drastic increase of ER-PM association in yeast and the extension of the ER tubules and redistribution of lysosomes to the cell periphery upon sterol depletion [67, 68]. Another contrast between malignant and non-malignant cells was the chol-dependent control of invadopodia *vs* focal adhesions and low invasion. These findings were consistent with the observation that the LDL-chol is delivered to the PM in close proximity of focal adhesions by the Rab8a-MyosinVb-actin pathway, favoring focal adhesion abundance and dynamics and promoting cell migration [69].

### Conclusion

Our data indicated the key contribution of chol-enriched surface domains in the control of invasion of breast cancer cell lines, providing new clues for the understanding of the molecular events underlying cellular mechanisms in breast cancer.

## Supplementary Information

Below is the link to the electronic supplementary material.

Supplementary file1 (DOCX 14963 KB)

## Data Availability

All data are available and the manuscript includes supplemental information submitted electronically.

## Abbreviations

A: MCF10A
AT: MCF10AT
AFM: Atomic force microscopy
CAIa: MCF10CAIa
chol: Cholesterol
cytoD: Cytochalasin D
ER: Endoplasmic reticulum
ECM: Extracellular matrix
MMP: Matrix metalloproteinase
mβCD: Methyl-β-cyclodextrin
PC: Phosphatidylcholine
PE: Phosphatidylethanolamine
PM: Plasma membrane
PS: Phosphatidylserine
SM: Sphingomyelin
