# Supplementary material for: Surface cholesterol-enriched domains specifically promote invasion of breast cancer cell lines by controlling invadopodia and extracellular matrix degradation
Source: Cell Mol Life Sci. 2022 Jul 12;79(8):417. doi: 10.1007/s00018-022-04426-8 (PMC9276565; doi:10.1007/s00018-022-04426-8)
Supplement: Supplementary file 1 — Supplementary file1 (DOCX 14963 KB) [file 18_2022_4426_MOESM1_ESM.docx]

**Supporting Information**

**Surface cholesterol-enriched domains specifically promote invasion of breast cancer cell lines by controlling invadopodia and extracellular matrix degradation**

*Mauriane Maja^1^, Danahe Mohammed^2^, Andra C. Dumitru^2^, Sandrine Verstraeten^3^, Maxime Lingurski^1^, Marie-Paule Mingeot-Leclercq^3^, David Alsteens^2^, Donatienne Tyteca^1^**

^1^CELL Unit and PICT Imaging Platform, de Duve Institute, UCLouvain, Brussels, Belgium

^2^Louvain Institute of Biomolecular Science and Technology (LIBST), UCLouvain, Ottignies-Louvain-la-Neuve, Belgium

^3^Cellular and Molecular Pharmacology Unit (FACM), Louvain Drug Research Institute, UCLouvain, Brussels, Belgium

***Corresponding author:** Donatienne Tyteca, CELL Unit, de Duve Institute, UCLouvain, B1.75.05, avenue Hippocrate, 75, B-1200 Brussels, Belgium. Phone: +32-2-764.75.91; Fax: +32-2-764.75.43; e-mail: [donatienne.tyteca@uclouvain.be](mailto:donatienne.tyteca@uclouvain.be)

**Materials and Methods**

**Western blotting.** Cells were washed in PBS, lysed in cold RIPA lysis buffer (150 mM NaCl, 0.5% sodium deoxycholate, 50 mM Tris, 0.1% SDS, 1% Triton X-100, 1 mM PMSF, cOmplete™ Protease Inhibitor Cocktail) for 25 min, then sonicated for 15 sec. Red blood cell ghosts were used as negative control and prepared as previously described [1, 2]. Cell lysates were mixed in 2% sample buffer (0.25 M Tris-HCl, pH 6.8, 10% SDS, 20% glycerol, 0.005% bromophenol blue) containing 5 mM Dithiothreitol (DTT) or not, and boiled for 5 min. Western blotting was performed as previously [3], except that samples were loaded on 3-10% SDS-PAGE gel.

**Thin Layer Chromatography.** Cells were treated with mβCD then washed in PBS. To lyse the cells, the cell suspension was diluted at 1x10^6^ cell/mL in 0.9% NaCl then submitted to 2 cycles of freezing/thawing at -80 °C and vortexed. To extract all lipids, 3 mL of ice-cold chloroform/methanol (2:1; v/v) was added to 1 mL of cell lysate. The solution was vortexed and centrifuged for 15 min at 3000 rpm at room temperature. The organic phase was evaporated and resuspended in 80 µL chloroform/methanol, separated by thin layer chromatography (TLC) in chloroform:methanol:15mM CaCl_2_ (65:35:8; v/v/v) and revealed by charring densitometry after staining with 10% cupric sulfate in 8% O-phosphoric acid. Lipids were identified with the standards and phosphatidylethanolamine (PE), phosphatidylcholine (PC), 1,2-dioleoyl-sn-glycero-3-phosphocholine (DOPC) and sphingomyelin (SM) band intensity was quantified using ImageJ/Fiji and expressed by reference to the sum of all band intensities.

**
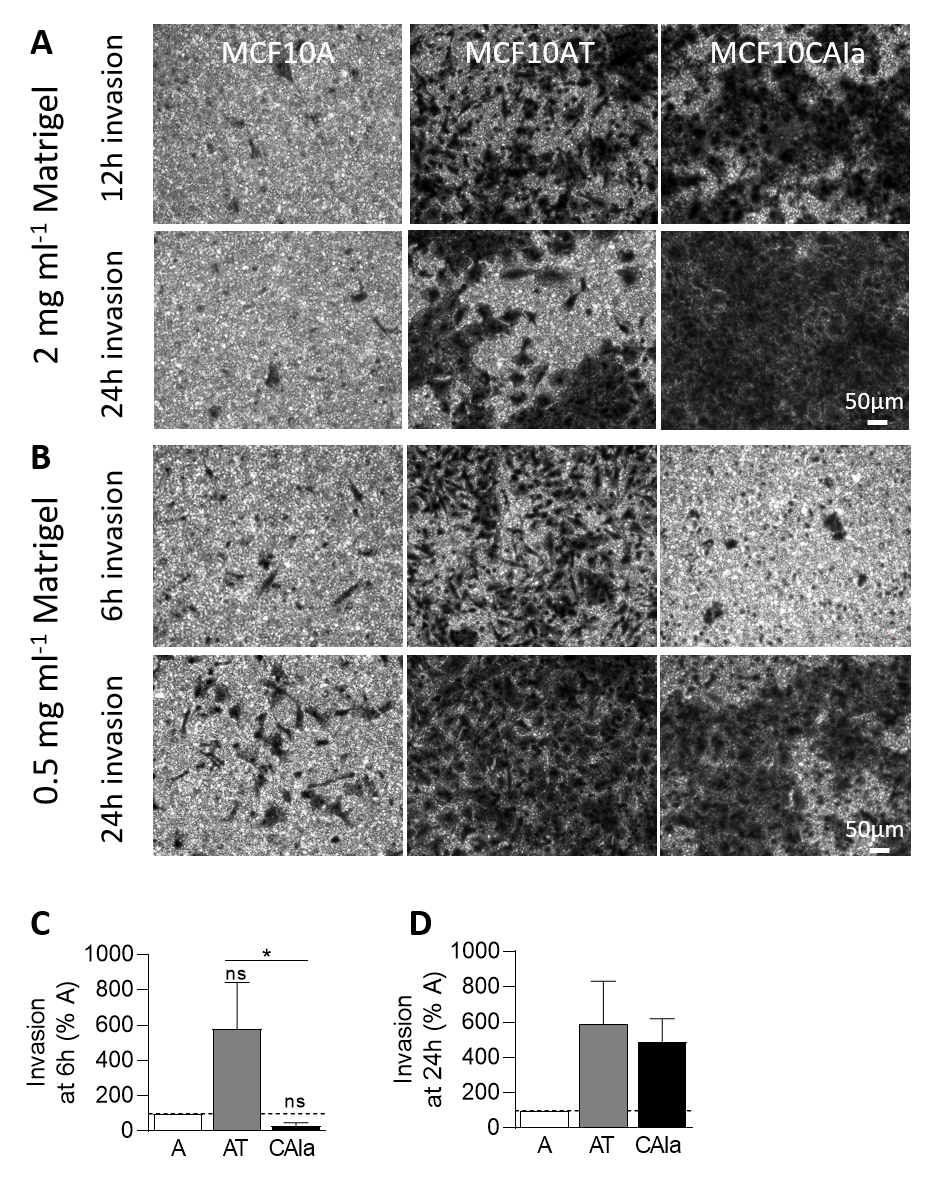
**

**Fig. S1** Effect of time and Matrigel concentration on invasion of the MCF10A cell line series. (A) Representative images of invasion of the 3 cell lines in Transwell with a dense (2 mg ml^-1^) Matrigel layer toward 10% serum for 12h and 24h. (B) Representative images of cell invasion potential in Transwell with a loose (0.5 mg ml^-1^) Matrigel layer toward 10% serum for 6h and 24h. (C-D) Quantification of invasion of the 3 cell lines in Transwell with a loose Matrigel layer toward 10% serum for 6h (C; n=3-4 Transwell from 4 independent experiments) or 24h (D; n=2 Transwell from 1 experiment). Kruskal-Wallis test followed by Dunn’s comparison test


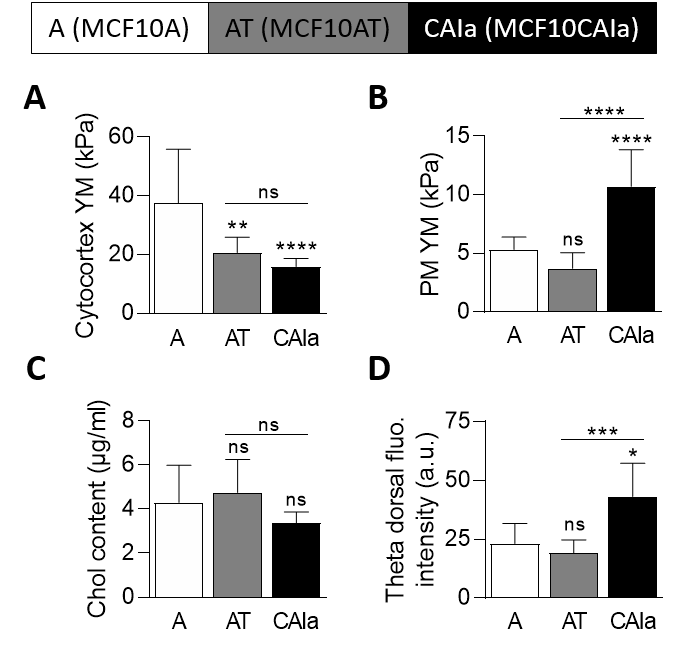


**Fig. S2** The MCF10A cell lines serie exhibits differential extents of cytocortex and plasma membrane stiffness, chol content and chol surface distribution. (A,B) Cytocortex (A) and PM (B) Young’s modulus of the 3 cell lines determined by AFM. Reproduced with permission [4] Copyright 2020, Advanced Science. (C) Total free chol content of the 3 cell lines assessed by Amplex Red assay (n=7-9 independent experiments). (D) Quantification of the Theta dorsal fluorescence intensity of the 3 cell lines plated on glass coverslips and labeled at 4 °C with the mCherry-Theta toxin fragment (4-10 cells from n=4-28 images from 3-8 independent experiments). Kruskal-Wallis test followed by Dunn’s comparison test.


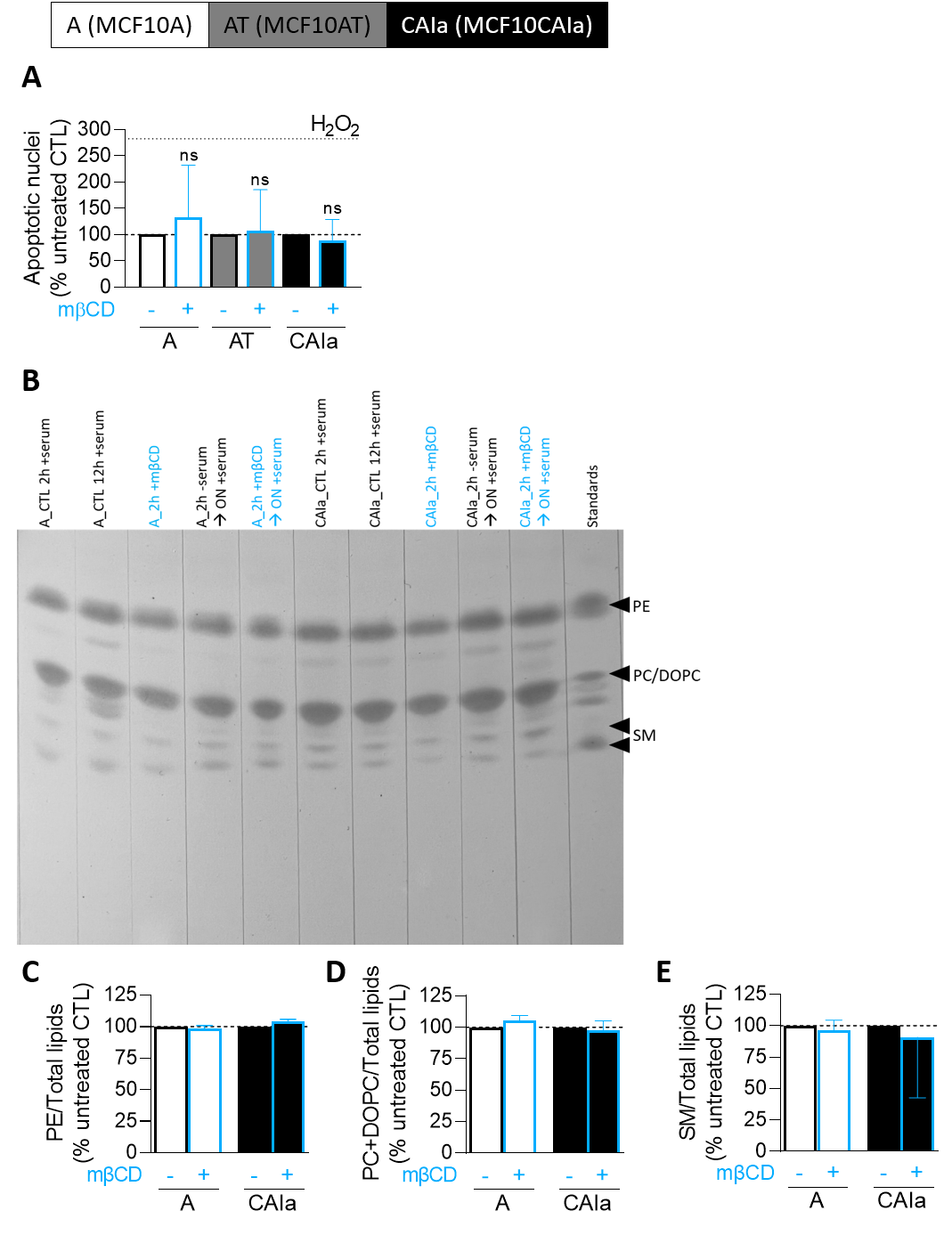


**Fig. S3** Cholesterol depletion by 2 mM methyl-β-cyclodextrin treatment does not induce apoptosis and does not extract sphingo- and phospholipids. (A) Quantification of the proportion of apoptotic nuclei in the 3 cell lines plated on glass coverslips, treated or not (-, black) with 2 mM mβCD for 2h (+, blue) then labeled with DAPI, visualized by confocal microscopy and quantified (n=1026-2418 nuclei from 3 independent experiments). Horizontal dotted line, 10 mM hydrogen peroxide (H_2_O_2_) used as positive control. Unpaired t test. (B-E) Thin layer chromatography on A and CAIa cell lysates. All cells were serum-starved for 2h, combined or not (black conditions) with 2 mM mβCD (blue conditions), then stimulated or not overnight (ON) with serum-containing medium. (B) Representative image. Arrowheads show the positions of phosphatidylethanolamine (PE), phosphatidylcholine (PC), 1,2-dioleoyl-sn-glycero-3-phosphocholine (DOPC) and sphingomyelin (SM; 2 bands) identified with the standards (n=1 experiment). (C-E) Quantification of PE (C), PC+DOPC (D) and SM (E) abundance in A and CAIa lysates treated or not with 2 mM mβCD (blue). Data represent the mean of 2h serum starvation followed or not by ON stimulation in serum conditions (n=1 experiment)


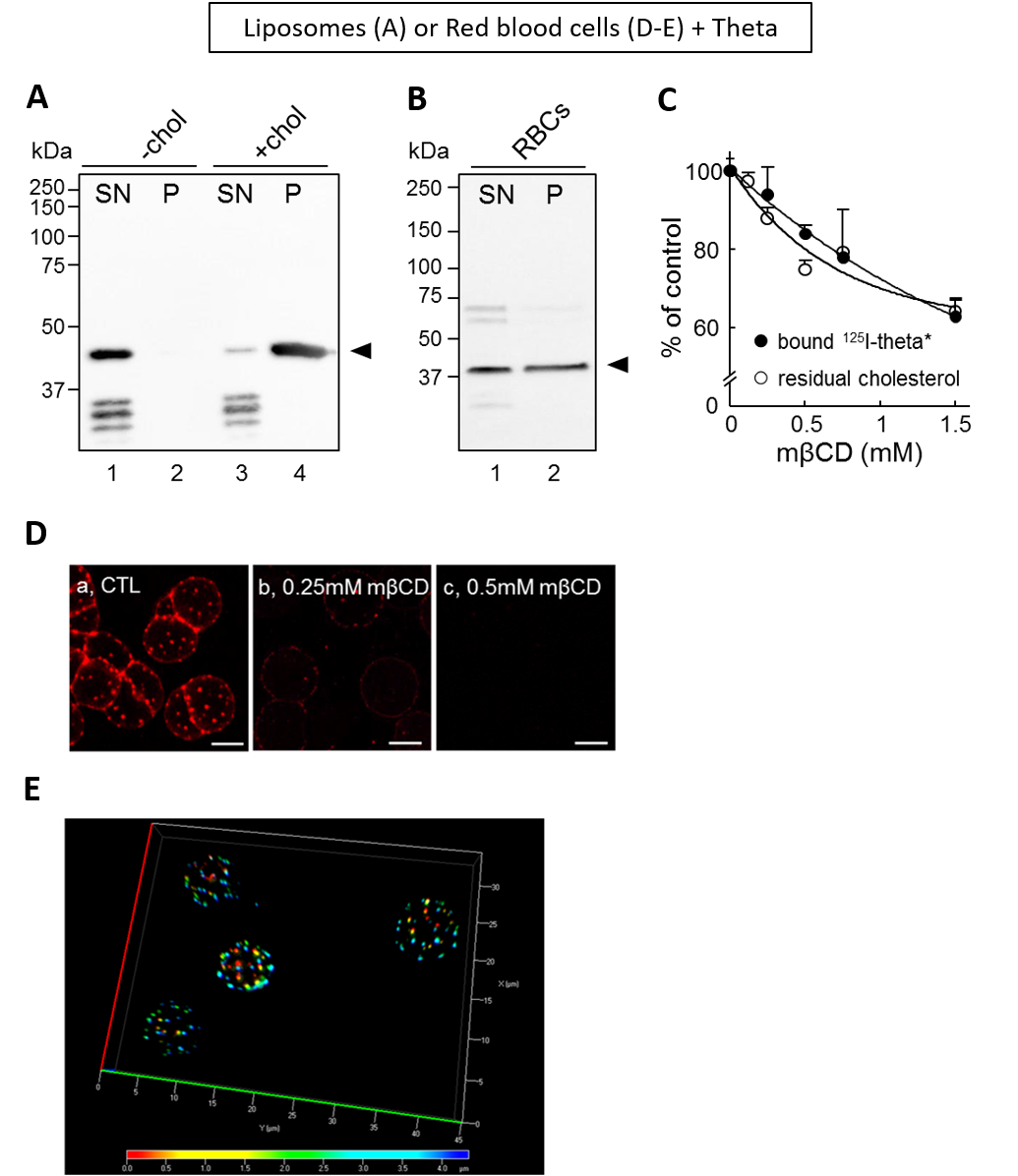


**Fig. S4** The Theta toxin fragment specifically binds to cholesterol and reveals cholesterol-enriched submicrometric domains on living spread red blood cells. (A,B) Specific binding of Theta to liposomes containing chol and to red blood cells (RBCs). Theta was incubated with multilamellar vesicles (MLVs; A) containing chol (+ chol; lanes 3 and 4) or not (- chol; lanes 1 and 2), or with freshly isolated RBCs in suspension (B). After centrifugation, pellets (P) containing MLVs (A) or RBCs (B) and supernatants (SN) were analyzed by Western blotting for the His-tag on Theta. Arrowheads correspond to the expected position of intact mCherry-Theta (~41kDa). (C) Correspondence between Theta binding and chol level in RBCs. Freshly isolated RBCs were either kept untreated or chol-depleted by mβCD prior to labeling in suspension with ^125^I-Theta mixed with 1µM cold Theta (in the continued presence of mβCD if appropriate). After washing by iterative centrifugation/resuspension, ^125^I-Theta bound to RBCs was measured and expressed as percentage of control cells (filled circles). Decrease of Theta binding closely parallels residual chol level (open circles). (D) Evidence for chol-enriched domains on living spread RBCs. Freshly isolated RBCs were either kept untreated (CTL; a) or chol-depleted by 0.25mM (b; - ~15% chol) or 0.5mM (c; - ~25% chol) mβCD at 37 °C, labeled in suspension with Theta in the continuous presence of mβCD, washed, attached-spread onto poly-L-lysine-coated coverslips and visualized by confocal microscopy at 20 °C. (E) Chol submicrometric domains are present on both sides of living spread RBCs. Fresh RBCs were labeled and spread and observed with wide-field fluorescence microscope. 3D-deconvolution and depth pseudo-coloration were then applied to visualize domain position in 3D (red corresponds to poly-L-lysine-free side, blue to poly-L-lysine-attached side). Adapted from [5]

**
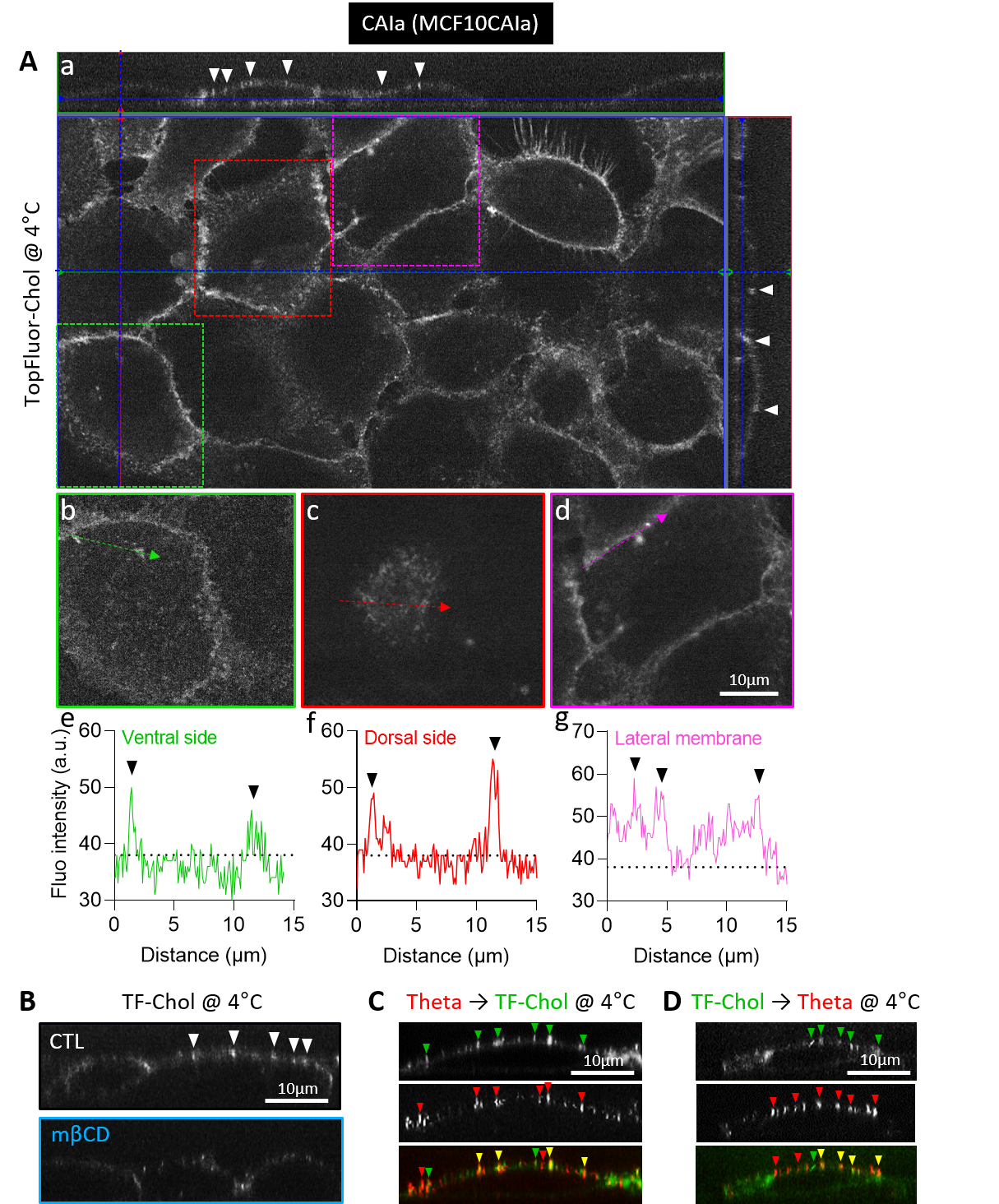
**

**Fig. S5** Plasma membrane insertion of TopFluor-Cholesterol reveals cholesterol-enriched submicrometric domains at the dorsal face of malignant CAIa cells that are decreased by methyl-β-cyclodextrin and largely correspond to the Theta-decorated domains. CAIa cells were plated on fibronectin-coated coverslips and then tested for surface chol distribution. (A) CAIa cells labeled at 4 °C with the chol analog TopFluor-Cholesterol. a, Representative image and X-Z reconstructions along the blue lines in the X-Y section. b-g, Insets showing 3 cells at ventral (green), dorsal (red) and lateral (purple) sections respectively and the corresponding fluorescence intensity profiles along the dotted lines (n=3 experiments). Arrowheads, chol-enriched domains. (B) CAIa treated or not with 2mM mβCD for 2h (blue) and labeled at 4 °C with the TopFluor-Cholesterol (TF-Chol). X-Z reconstructions of confocal images are shown (n=2 experiments). Arrowheads, dorsal chol-enriched domains. (C,D) CAIa cells sequentially labeled at 4 °C with the mCherry-Theta toxin fragment specific to endogenous chol then with the TopFluor-Cholesterol (C) or vice versa (D) (n=2 experiments). Arrowheads, dorsal chol-enriched domains labeled with the TopFluor-Cholesterol (green), with the mCherry-Theta toxin fragment (red) or with both probes (yellow)

**
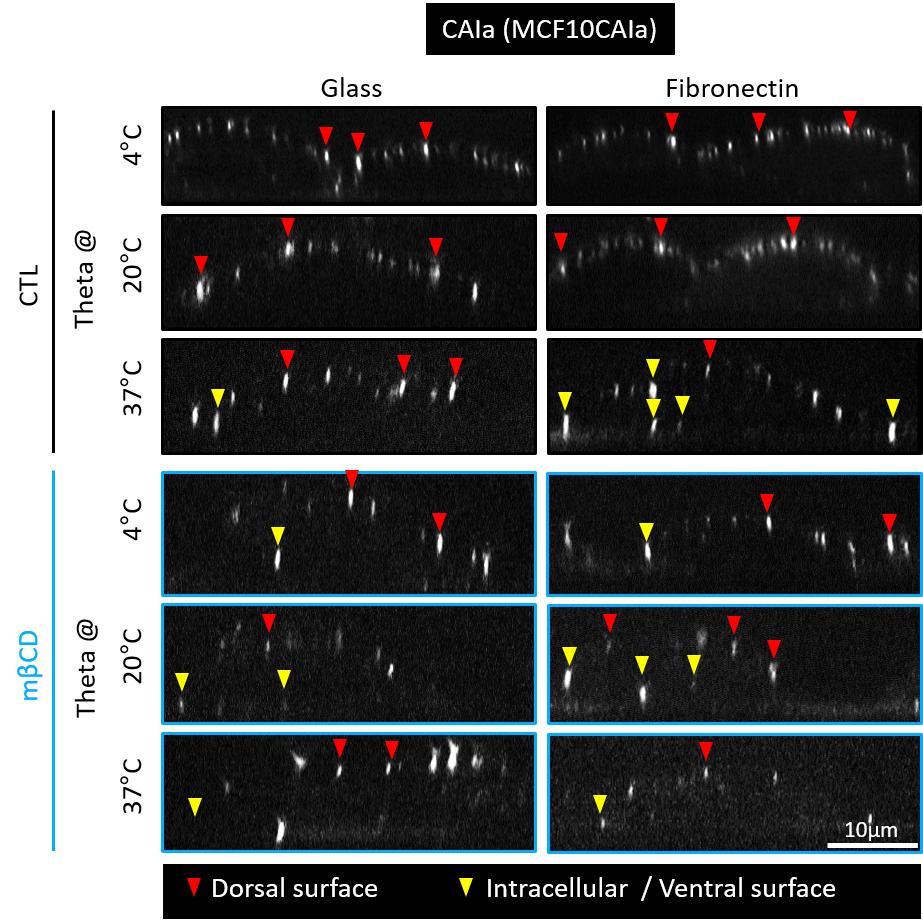
**

**Fig. S6** Cholesterol depletion removes a dorsal surface cholesterol pool that can be internalized and reach the ventral face. MCF10CAIa were plated on glass or fibronectin-coated coverslips, treated with 2 mM mβCD for 2h (blue) and labeled at the indicated temperature with the mCherry-Theta toxin fragment as in Figure 2I. X-Z reconstructions of confocal images

**
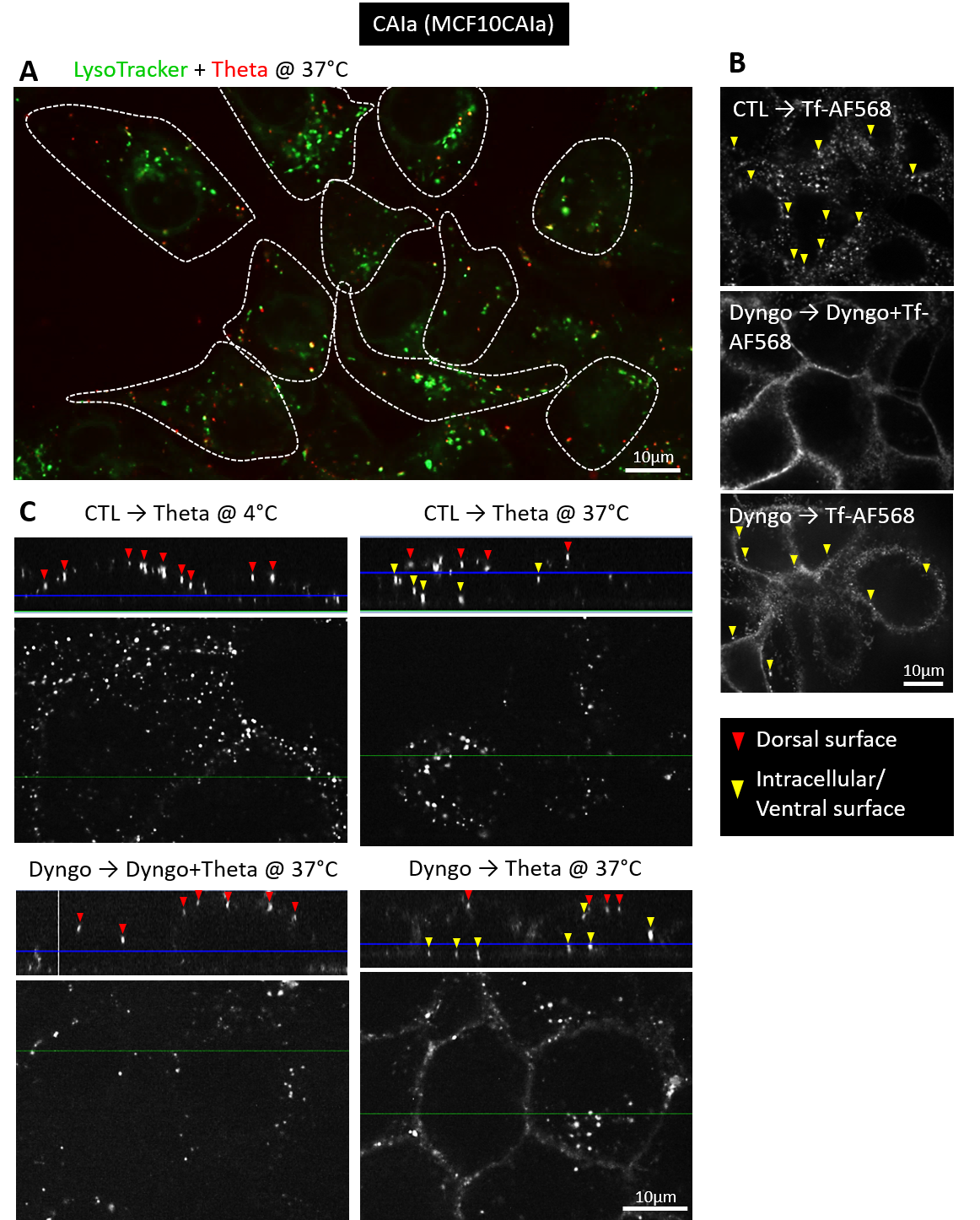
**

**Fig. S7** Large colocalization of Theta with late endosomes/lysosomes and impairment of cholesterol-enriched domain redistribution to the ventral side upon inhibition of endocytosis in malignant CAIa cells. (A) Extension of Fig. 2J showing CAIa cells colabeled at 37 °C with the mCherry-Theta toxin fragment and the LysoTracker. (B,C) Inhibition of endocytosis in CAIa cells plated on fibronectin-coated coverslips, treated or not with 20 µM Dyngo4a, a dynamin inhibitor, then incubated at 37 °C with the Alexa568-transferrin (B; Tf-AF568 used as positive control) or with the mCherry-Theta toxin fragment (C) in the continuous presence or not of Dyngo4a and analyzed by confocal microscopy. Red arrowheads, chol-enriched domains at the dorsal side; yellow arrowheads, chol-enriched domains or Tf-positive structures inside the cells or at ventral face


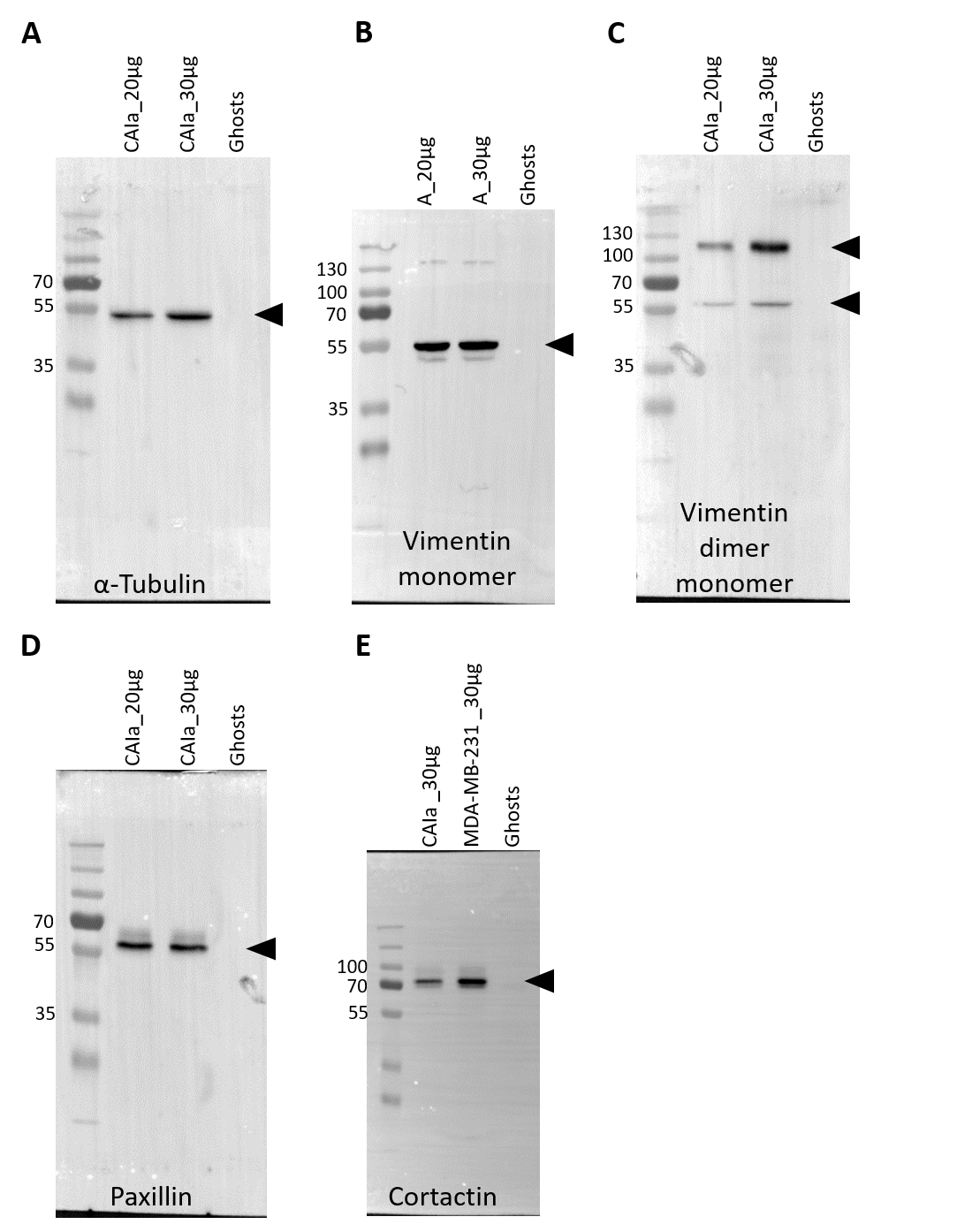


**Fig. S8** Specificity of anti-α-Tubulin, -Vimentin, -Paxillin and -Cortactin antibodies. Western blotting against α-Tubulin (A), Vimentin (monomer in A and mainly dimer in CAIa cells; B and C respectively), Paxillin (D) and Cortactin (E) on A (B), CAIa (A,C,D,E) and/or MDA-MB-231 (E) cell lysates. Erythrocyte ghosts were used as negative controls


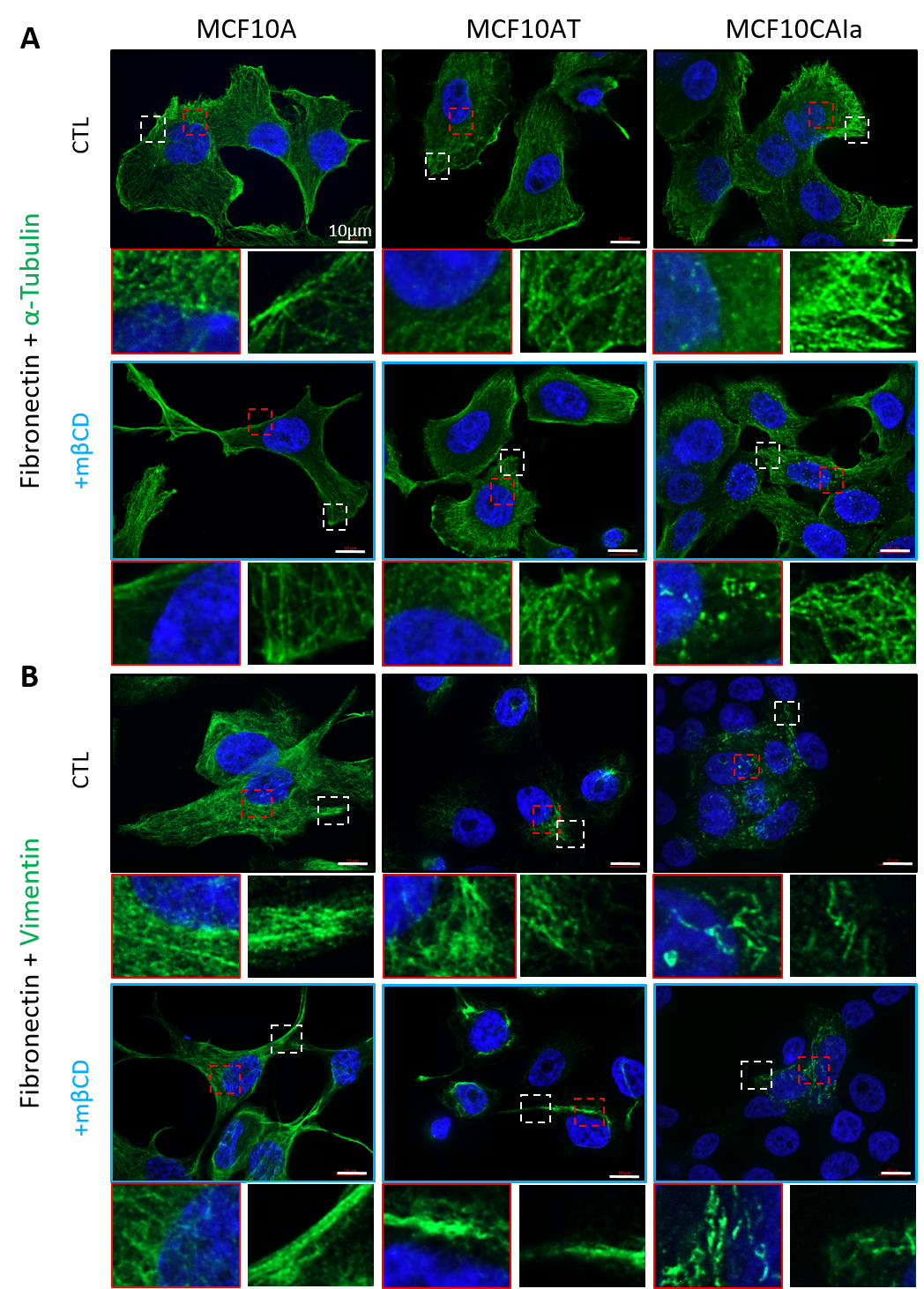


**Fig. S9** Preservation of microtubule and intermediate filament distribution upon cholesterol depletion. The 3 cell lines were plated on fibronectin-coated coverslips, treated or not with 2 mM mβCD for 2h (blue), immunolabeled with anti-α-Tubulin (A) or anti-Vimentin (B) and visualized by confocal microscopy. Nuclei stained with Hoechst in blue. Red insets, perinuclear regions; White insets, peripheral regions

**
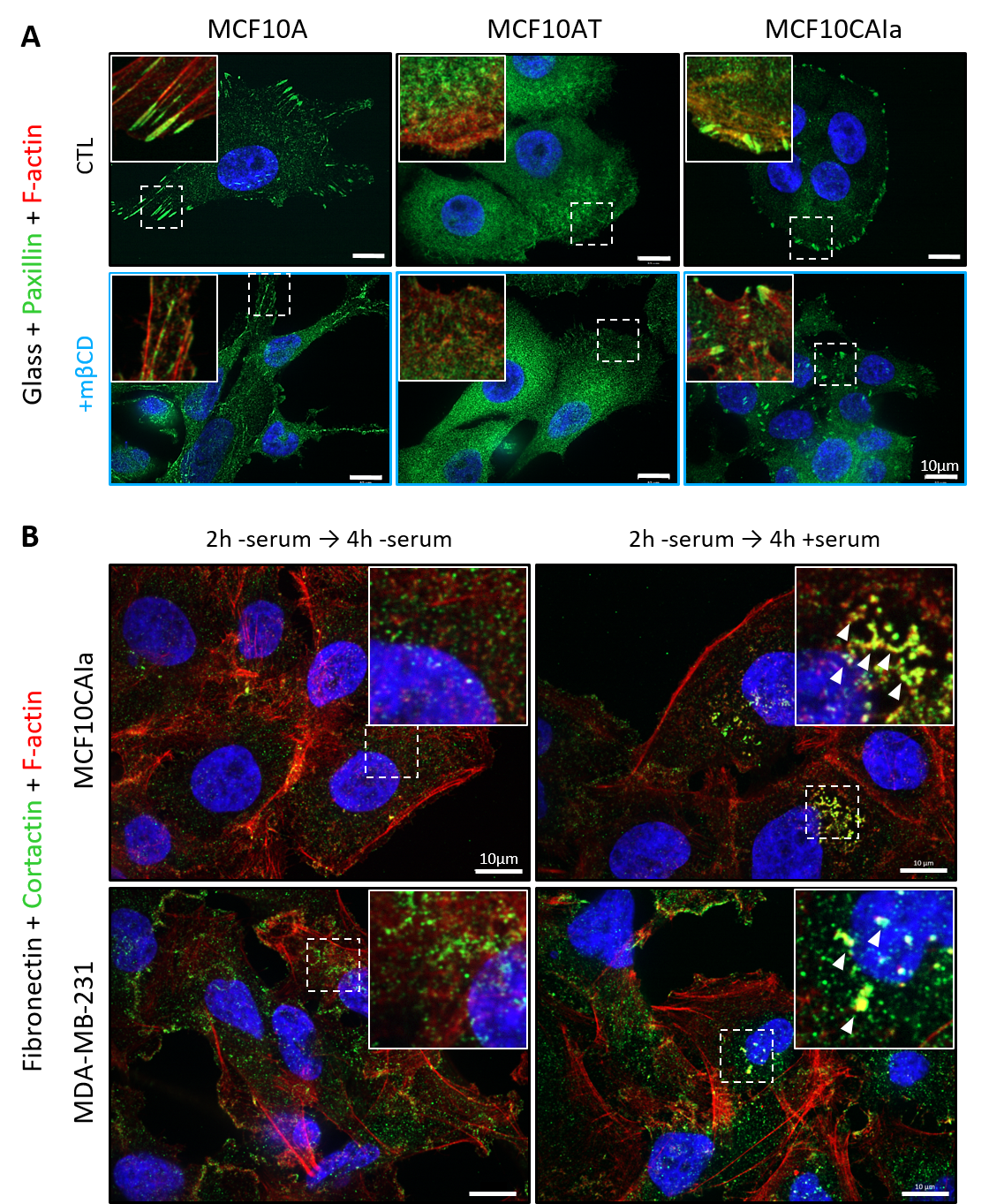
**

**Fig. S10** Cholesterol depletion impairs focal adhesions specifically in normal cells and serum starvation followed by reincubation in serum-containing medium stimulates the formation of invadopodia. (A) Confocal images of the 3 cell lines plated on glass coverslips, treated or not with 2 mM mβCD for 2h and immunolabeled with anti-Paxillin together with F-Actin (Phalloidin) and nuclei (Hoechst). Insets show Paxillin-positive focal adhesions. (B) Confocal images of CAIa and MDA-MB-231 plated on fibronectin-coated coverslips, serum starved for 2h then stimulated 4h with serum containing medium to allow reformation of invadopodia and immunolabeled with anti-Cortactin together with F-actin (Phalloidin) and nuclei (Hoechst)

**
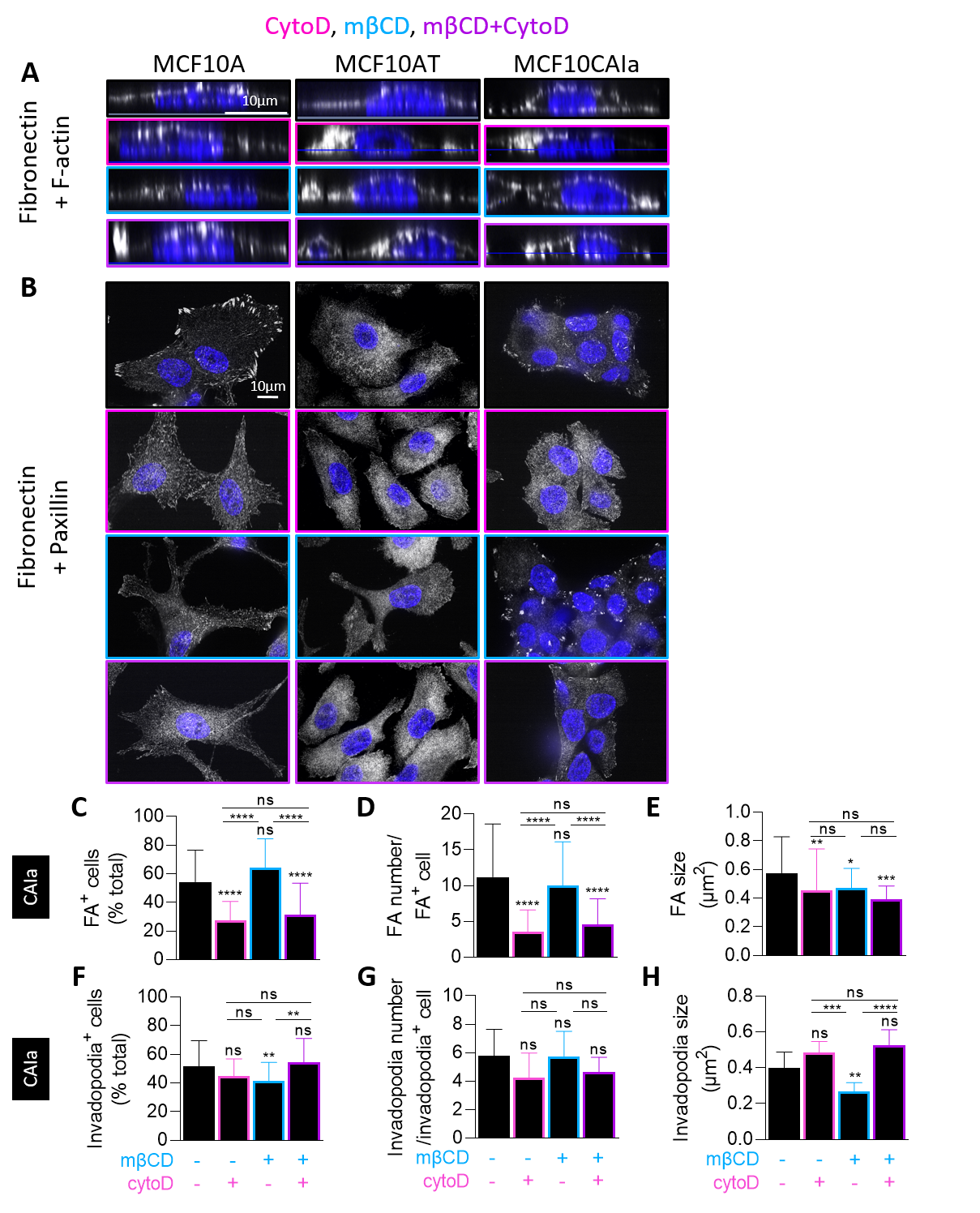
**

**Fig. S11** Effect of combined cholesterol depletion and actin polymerization inhibition on actin cytoskeleton, focal adhesions and invadopodia in the MCF10A cell line series. Cell lines were treated with 2 mM mβCD (blue) in combination (purple) or not with 0.5 µM cytoD (pink) for 2h and then tested for confocal microscopy. (A) X-Z reconstructions of confocal images of the 3 cell lines plated on fibronectin-coated coverslips, treated or not with mβCD in combination with cytoD and then labeled with Phalloidin (F-actin) and nuclei (Hoechst). (B) Confocal images of the 3 cell lines plated on fibronectin-coated coverslips, treated or not with mβCD in combination with cytoD and immunolabeled with anti-Paxillin. Nuclei stained with Hoechst in blue. (C-H) Quantification of the number of cells presenting focal adhesions (C), the number of focal adhesions per cell (D), the focal adhesion size (E; n=33-35 images [C] and n=33-84 cells [D,E] from 2-5 independent experiments), the number of cells presenting invadopodia (F), the number of invadopodia per cell (G) and the invadopodia size (H; n=8-65 images from 2 independent experiments) of CAIa plated on fibronectin-coated coverslips, treated with mβCD in combination or not with cytoD and immunolabeled with anti-Paxillin (C-E) or anti-Cortactin (F-H). One-Way ANOVA test followed by Tukey’s comparison test (C-F) and Kruskal-Wallis test followed by Dunn’s comparison test (G,H)

**
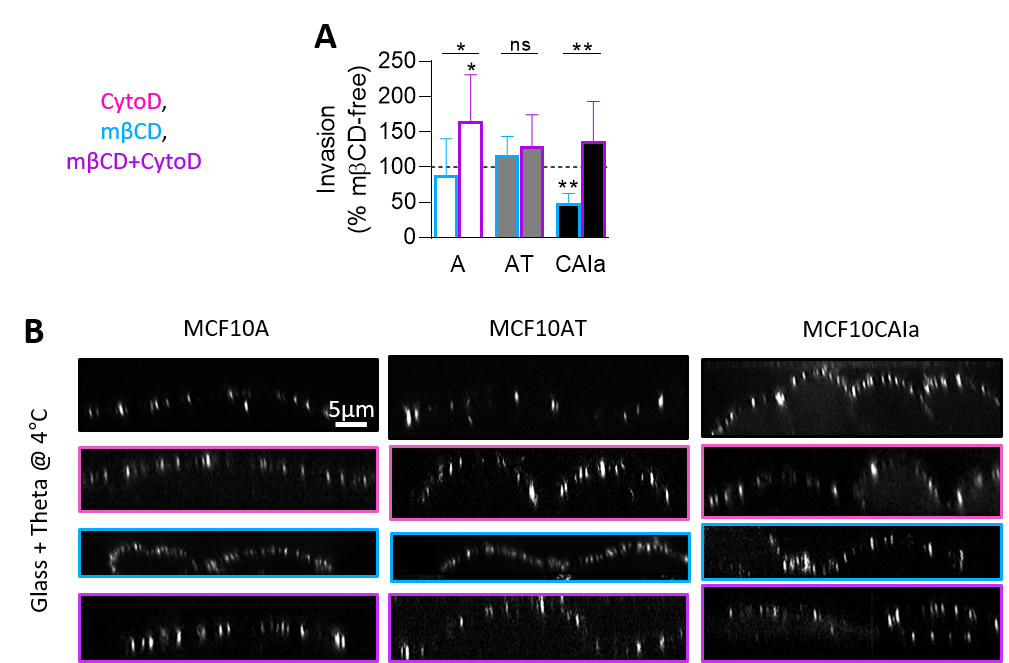
**

**Fig. S12** Effect of combined cholesterol depletion and actin polymerization inhibition on invasion and surface cholesterol distribution in the MCF10A cell line series. Cell lines were treated with 2 mM mβCD (blue) in combination (purple) or not with 0.5 µM cytoD (pink) for 2h and then tested for invasion and confocal microscopy. (A) Quantification of invasion of the 3 cell lines in Transwell with a dense Matrigel layer toward 10% serum for 6-12h. Data are expressed in percentage of mβCD-free conditions (n=5-9 Transwell from 2-3 independent experiments). Mann-Whitney test and Wilcoxon signed-rank test. (B) X-Z reconstructions of confocal images of the 3 cell lines plated on glass coverslips, treated or not with mβCD in combination with cytoD and then labeled at 4 °C with the mCherry-Theta toxin fragment

**
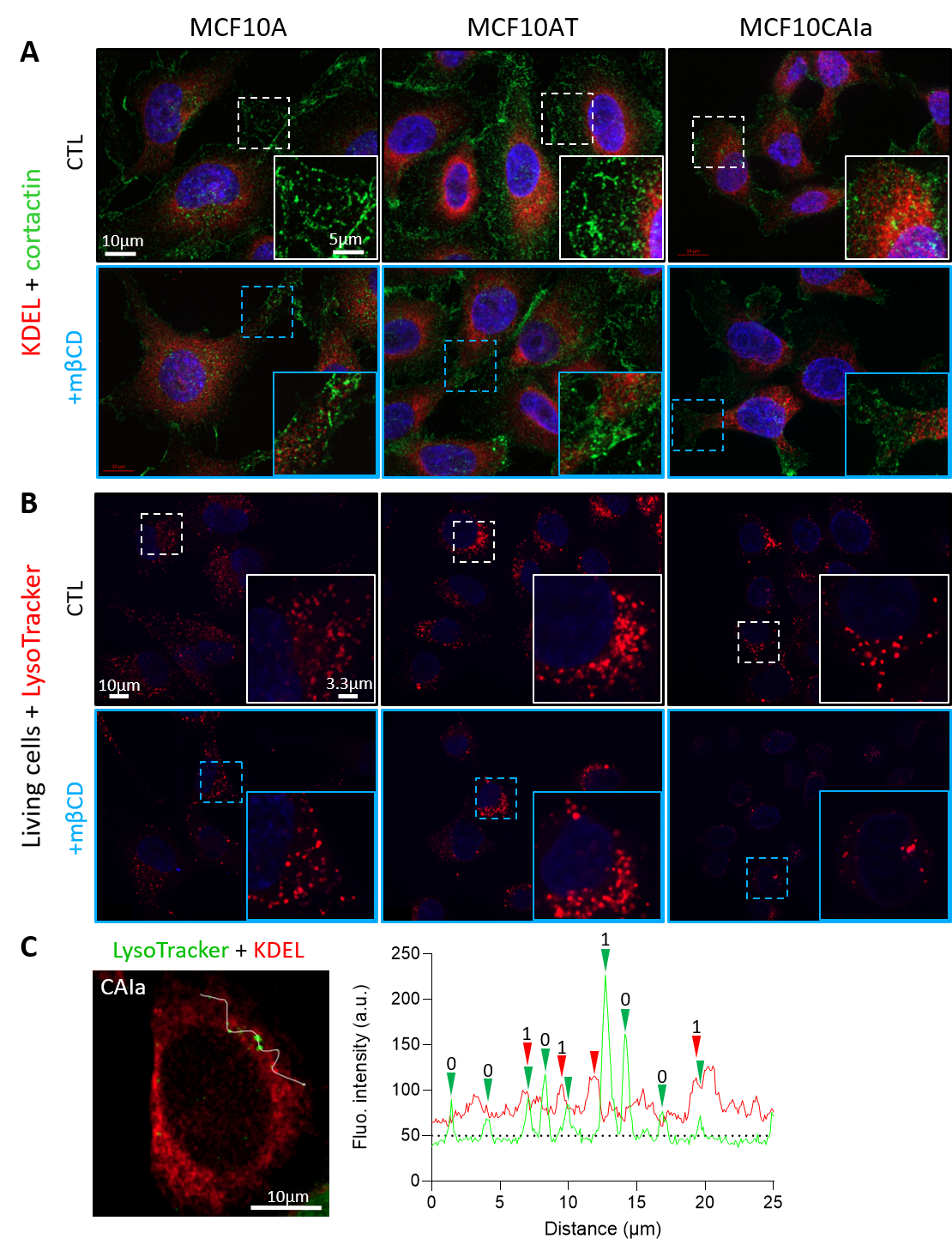
**

**Fig. S13** Effect of cholesterol depletion on endoplasmic reticulum spreading towards the cell ventral side and lysosome distribution. The three cell lines were plated on fibronectin-coated coverslips, treated or not with 2 mM mβCD for 2h (blue) and then tested for confocal microscopy. (A) Coimmunolabeling with anti-KDEL (ER) and anti-Cortactin (to reveal invadopodia at the ventral side). (B) Labeling at 37 °C with the LysoTracker. Nuclei stained with Hoechst. Insets show ER network spreading near invadopodia sites (A) and lysosome abundance and distribution (B). (C) Representative fluorescence intensity profile along the line on the CAIa cell colabeled with anti-KDEL and the LysoTracker. Attribution of scores for close (=1) or loose (=0) contacts between green LysoTracker and red KDEL signals

**References**

1. Prausnitz MR, Lau BS, Milano CD, Conner S, Langer R, Weaver JC. A quantitative study of electroporation showing a plateau in net molecular transport. Biophys J. 1993;65(1):414-22. Epub 1993/07/01. doi: 10.1016/S0006-3495(93)81081-6. PubMed PMID: 7690262; PubMed Central PMCID: PMCPMC1225736.

2. Pollet H, Cloos AS, Stommen A, Vanderroost J, Conrard L, Paquot A, et al. Aberrant Membrane Composition and Biophysical Properties Impair Erythrocyte Morphology and Functionality in Elliptocytosis. Biomolecules. 2020;10(8). Epub 2020/08/06. doi: 10.3390/biom10081120. PubMed PMID: 32751168; PubMed Central PMCID: PMCPMC7465299.

3. Dumitru AC, Stommen A, Koehler M, Cloos AS, Yang J, Leclercqz A, et al. Probing PIEZO1 Localization upon Activation Using High-Resolution Atomic Force and Confocal Microscopy. Nano Lett. 2021;21(12):4950-8. Epub 2021/06/15. doi: 10.1021/acs.nanolett.1c00599. PubMed PMID: 34125553.

4. Dumitru AC, Mohammed D, Maja M, Yang J, Verstraeten S, Del Campo A, et al. Label-Free Imaging of Cholesterol Assemblies Reveals Hidden Nanomechanics of Breast Cancer Cells. Adv Sci (Weinh). 2020;7(22):2002643. Epub 2020/11/27. doi: 10.1002/advs.202002643. PubMed PMID: 33240781; PubMed Central PMCID: PMCPMC7675049.

5. Carquin M, Conrard L, Pollet H, Van Der Smissen P, Cominelli A, Veiga-da-Cunha M, et al. Cholesterol segregates into submicrometric domains at the living erythrocyte membrane: evidence and regulation. Cell Mol Life Sci. 2015;72(23):4633-51. Epub 2015/06/17. doi: 10.1007/s00018-015-1951-x. PubMed PMID: 26077601.
